# Temporal Gene Expression of the Cyanobacterium *Arthrospira* in Response to Gamma Rays

**DOI:** 10.1371/journal.pone.0135565

**Published:** 2015-08-26

**Authors:** Hanène Badri, Pieter Monsieurs, Ilse Coninx, Robin Nauts, Ruddy Wattiez, Natalie Leys

**Affiliations:** 1 Expert Groups for Molecular and Cellular Biology and Biosphere Impact Studies, Belgian Nuclear Research Centre SCK•CEN, Mol, Belgium; 2 Proteomics and Microbiology Group, Research Institute for Biosciences, University of Mons, Mons, Belgium; Louisiana State University and A & M College, UNITED STATES

## Abstract

The edible cyanobacterium *Arthrospira* is resistant to ionising radiation. The cellular mechanisms underlying this radiation resistance are, however, still largely unknown. Therefore, additional molecular analysis was performed to investigate how these cells can escape from, protect against, or repair the radiation damage. *Arthrospira* cells were shortly exposed to different doses of ^60^Co gamma rays and the dynamic response was investigated by monitoring its gene expression and cell physiology at different time points after irradiation. The results revealed a fast switch from an active growth state to a kind of 'survival modus' during which the cells put photosynthesis, carbon and nitrogen assimilation on hold and activate pathways for cellular protection, detoxification, and repair. The higher the radiation dose, the more pronounced this global emergency response is expressed. Genes repressed during early response, suggested a reduction of photosystem II and I activity and reduced tricarboxylic acid (TCA) and Calvin-Benson-Bassham (CBB) cycles, combined with an activation of the pentose phosphate pathway (PPP). For reactive oxygen species detoxification and restoration of the redox balance in *Arthrospira* cells, the results suggested a powerful contribution of the antioxidant molecule glutathione. The repair mechanisms of *Arthrospira* cells that were immediately switched on, involve mainly proteases for damaged protein removal, single strand DNA repair and restriction modification systems, while *recA* was not induced. Additionally, the exposed cells showed significant increased expression of *arh* genes, coding for a novel group of protein of unknown function, also seen in our previous irradiation studies. This observation confirms our hypothesis that *arh* genes are key elements in radiation resistance of *Arthrospira*, requiring further investigation. This study provides new insights into phasic response and the cellular pathways involved in the radiation resistance of microbial cells, in particularly for photosynthetic organisms as the cyanobacterium Arthrospira.

## Introduction

*Arthrospira* is a motile filamentous planktonic non-N_2_-fixing cyanobacterium. It grows naturally in alkaline lake water environments in regions with strong sunlight and high temperature [1]. *Arthrospira* are also cultivated on industrial scale for animal and human consumption and has gained considerable popularity as natural microbial dietary supplement in the health food industry [2]. Its valuable nutritious components include essentially fatty acids such as omega-3, pigments such as carotenes and phycocyanin, and minerals such as iron [3, 4]. Due to its high nutritive value and claimed anti-oxidant and anti-inflammatory activities [5], *Arthrospira* is considered as a promising nutraceutical with applications on earth and in space [6].

The resilience of *Arthrospira* cells to extreme environmental conditions was reported. It has the ability to grow in highly alkaline environments [7], environments with high salt concentration: 1.5 higher than sea, ranging between 20 to 30 g L^-1^[8], at extreme temperatures lower than 15°C and higher than 45°C [9], and at high light intensities as 500 μE/m^2^/s [10]. It is also able to resist ionising radiation from UV and gamma rays, as well as charged particles [11, 12]. The response of *Arthrospira* to such environmental stresses has mainly been investigated based on morphological and physiological traits [13]. Recently, we were the first to study the damage of radiation stress (i.e. high doses of gamma rays) at molecular level, using a combination of genomic, transcriptomic and proteomic data [12]. Cells exposed to high doses of 3200 or 5000 Gy of gamma rays, causing significant cell damage, showed organised shutdown of photosynthesis and carbon fixation, decreased pigment, lipid and secondary metabolite synthesis; and induced thiol-based antioxidant systems, photo-sensing and signalling pathways.

Here we extended our molecular research of the radiation resistance of *Arthrospira*, to lower and less lethal doses of gamma rays (800 Gy and 1600 Gy, in addition to 3200 Gy) received in short exposure time, thereby allowing gene expression profiling immediately after the exposure, and at different time points during recovery after irradiation. We included also some additional biochemical measurements. This approach allowed us to map the dynamics of the response and related metabolic pathways in function of time. Thus, this study aims to elucidate the molecular mechanisms of *Arthrospira* cells to resist to and recover from radiation exposure.

## Materials and Methods

### Strain and culture conditions

The strain of *Arthrospira sp*. PCC 8005 was originally obtained from the Pasteur Culture Collection, sub-cultured, and maintained as active cultures in the lab at SCK•CEN. Three independent lab cultures (i.e. three biological replicates, n = 3) were propagated and used separately for irradiation. They were grown aerobically in 1000 ml Zarrouk-UBP medium in 2000 ml Erlenmeyers [14] on a rotatory shaker 120 rpm in an incubator at 30°C illuminated with a photon flux density of ~ 42 μE m^-2^ s^-1^ provided by three Osram daylight tubes (Binder KBW 400), to mid-exponential growth phase corresponding to an optical density of OD_750nm_ ~1 and a Zarrouk-UBP medium pH of ca. 10.5. Each of the 3 separate 1000 ml cultures was then divided in three aliquots (ca. 300 ml each) to be used for the three different irradiation experiments (three doses: 800 Gy, 1600 Gy and 3200 Gy). And each 300 ml aliquot, was further divided into two (ca. 150 ml each), to split the cultures into irradiation samples and non-irradiated controls in different flasks (500 ml CELLSTAR Filter cap cell, Greiner Bio-One, Vilvoorde, Belgium). Thus for each irradiation dose, three independent cultures were irradiated, and in parallel of each irradiated sample, an equal non-irradiated sample was kept.

### Irradiation conditions

The irradiation was performed using BRIGITTE facility at the Belgian Reactor N°2 (BR2) of SCK•CEN. The irradiation was done inside a closed canister under water, surrounded by ten ^60^Co gamma rays sources (Photon energy of 1.33 MeV and 1.17 MeV). *Arthrospira* cultures were irradiated with different doses of gamma rays, i.e. 800 Gy, 1600 Gy and 3200 Gy, using a constant dose rate of 20 000 Gy h^-1^. The time required for irradiation was dose-dependent, i.e. 2.4 minutes for 800 Gy, 4.8 minutes for 1600 Gy and 9.6 minutes for 3200 Gy. Irradiation was done on aerobic liquid cultures, containing active planktonic filaments suspended in ca. 150 ml liquid Zarrouk-UBP medium, in plastic culture flasks (CELLSTARFilter Cap Cell; 500 ml Cell Culture Flasks). During irradiation, the cultures were in the dark, and the temperature inside the irradiation canister was automatically monitored and ranged between 26–27°C. In parallel, three other cultures were maintained at the same conditions (also in the dark) but outside the irradiation facility, as non-irradiated controls. Samples were immediately shielded on ice after irradiation, at the irradiation facility, before transport to the lab for further processing. Some aliquots were used immediately after irradiation at T(0H) for regrowth (1 ml aliquot) and measurement of chlorophyll fluorescence (2 ml aliquot). Another part of the samples was centrifuged at 4°C (10 000 g, 20 min) and the obtained cell pellets were flash frozen in liquid nitrogen, and further conserved at-80°C, for molecular and biochemical analysis, including mRNA profiling (30 ml aliquot), glutathione content (15 ml aliquot) and pigment content (2 ml aliquot) quantification.

The remaining part of the sample was put back into the incubator (at the same conditions as mentioned above) after irradiation for recovery and was harvested 2h and 5h after irradiation.

### Post-irradiation recovery and proliferation analysis

In order to investigate the ability of *Arthrospira* filaments to recover after irradiation, 1 ml inoculation of 1% (v/v) from irradiated and non-irradiated samples (at OD_750_ ~1), including three independent biological replicates per test condition (n = 3), was done in 100 ml fresh Zarrouk-UBP medium, and incubated for growth at the same conditions as cited above. The growth was followed by optical density OD_750nm_ measurements, *i*.*e*. absorbance measurement at 750 nm, every three days using a spectrophotometer (AquaMate, Unicam, Cambridge, UK). The proliferation curves were plotted as OD_750nm_ increase versus time.

### Photosystem II quantum yield

Immediately after irradiation, a 2 ml aliquot was taken from all the irradiated cultures and their respective controls (at OD_750_ ~1), including three independent biological replicates per test condition (n = 3), and further dark adapted at room temperature for 15 min before photosynthetic potential measurements. Photosynthetic potential was measured by Phycobilisome and chlorophyll A fluorescence of Photosystem II (PSII) using the DUAL PAM 100 (Waltz-GmbH Effeltrich Germany). First, the cells were exposed to a weak modulated red light (ML) (635 nm, 3 μE.m^-2^.s^-1^) (which is too low in intensity to induce any photosynthetic activity), and minimum fluorescence was determined (F0). Next, the cells were exposed to a high intensity light flash or saturating pulse of red light (635 nm, 8000 μE.m^-2^.s^-1^) with short duration (0.8 s) (which is too sudden and short to induce any photosynthetic activity) and maximum fluorescence in dark adapted state (Fm) was determined. From those measurements, the ratio Fv/Fm was then calculated where the variable fluorescence Fv is equal to Fm—F0. Fv/Fm represents the maximum potential quantum efficiency of Photosystem II. An Fv/Fm value around of 0.6 is the approximate optimal value for *Arthrospira* species [15], with lower values indicating cell stress [16].

### Pigments analysis

Immediately after exposure to gamma rays, aliquots of two ml were taken from all irradiated and control cultures (at OD_750nm_ ~1), including three independent biological replicates per test condition (n = 3), for pigment analysis. Cells were collected by centrifugation (10000 g, 15 min, RT) (Microcentrifuge 5418R, Eppendorf), and cell pellets were stored at-80°C until analysis (ca. 2 days). Later, frozen cell pellets were dried overnight using a freeze-dryer (Lyovac GT 2, GEA Lyophil GmbH), and the absolute dry weight was determined. Next, the pellets were re-suspended using 1 ml of 0.05 M Na_2_HPO_4_ at pH 7, and, five cycles of freezing in liquid nitrogen and thawing at 37°C were performed, to crack the cells and release the phycobiliproteins containing phycocyanin and allophycocyanin pigments. In order to achieve total extraction, an additional treatment of 30 min at 37°C with 100 μl of lysozyme at a final concentration of 100 mg ml^-1^ was performed. Next, the suspension was centrifuged (13000 g, 10 min, RT) (Microcentrifuge 5418R, Eppendorf), the pellet was kept apart for further analysis and the supernatant containing the water soluble pigments phycocyanin (PC) and allophycocyanin (APC), was measured for absorbance at wavelengths 615 and 652 nm, using a photospectrometer (Unicame, Aquamat). The concentration of PC and APC were calculated according to following formula: $PC=\frac{\left(\text{OD}615)-0.474(\text{OD}652\right)}{5.34}$ and $APC=\frac{\left(\text{OD}652)-0.208(\text{OD}615\right)}{5.09}$ [17]. Then three washing steps were done on the remaining pellet using 1 ml of 0.05 M Na_2_HPO_4_ at pH 7, followed by a chlorophyll extraction step using 1 ml of 100% methanol as organic solvent. Additional mechanic treatment by sonication (3 cycles of 10 s, amplitude 30%, 1 pulse per second; Vibra cells) was performed to enhance total chlorophyll extraction. The suspension was centrifuged (13000 g, 10 min, at 4°C) (Microcentrifuge 5418R, Eppendorf) and the supernatant was measured via absorbance spectrophotometry at a wavelength of 665 nm, using a photospectrometer (Unicame, Aquamat). Chlorophyll (Chl) concentration was calculated then according to following formula: $Chl=\frac{\text{OD}665}{74.5}$ [17], where 74.5 ml.mg^-1^.cm^-1^ is the extinction coefficient of Chl A at 665nm in absolute methanol. The pigment concentrations were calculated to pigment weight (mg) per biomass dry weight (g) (w/w) and the results from irradiated samples were plotted as percentage of their representative non-irradiated control (which was put at 100%).

### Glutathione measurement

Aliquots of fifteen ml were taken from all irradiated and control samples (at OD750 ~1), including three independent biological replicates per test condition (n = 3), and cells were collected via centrifugation (10 000 g, 20 min) using (Avanti J- 26XP; Beckman Coulter, Suarlée, Belgium). The cells were transferred into 1.5 ml collection tubes and the wet biomass weight was determined for each sample. The cells were put in liquid nitrogen and shredded via bead-beating during 3.5 min at 30 Hz using two tungsten carbide beads (Ball Mill Mixer MM400, Retsh, Germany). Further extraction was performed by adding 400 μl of 200 mM HCl solution to the cells. Then, the samples were centrifuged at 4°C (15 min, 13200 rpm) (Microcentrifuge 5418R, Eppendorf). Aliquots of 300 μl from each sample were mixed to 30 μl NaH_2_PO_4_ buffer (pH 5.6). The pH was adjusted in the range of 3.5–5.0 by adding 200 mM NaOH. During the entire extraction procedure the samples were kept on ice. The final reactions for glutathione measurements were done at room temperature. The glutathione specific measurement used in this study is based on the reduction of Ellman reagent (DTNB, 5,5_-dithiobis-2-nitro-benzoic acid) by the catalytic action of glutathione (GSH or GSSG) in the presence of NADPH and glutathione reductase. The Ellman's reagent will react with the sulfhydryl groups (= thiol groups) of the glutathione, releasing the soluble chromophore 2-nitro-5-thiobenzoate, and the absorbance of the chromophore 2-nitro-5-thiobenzoate is read at 412 nm. By adding the glutathione reductase (GR) and NADPH into the reaction of GSH and DTNB, the color yield is much higher (compared to color yield in the absence of GR), and the rate of extra color development is depending specifically on the initial concentration of the total glutathione in the mixture, which makes the current method specific for quantifying glutathione. Reactions were performed in the presence of 100 μl phosphate buffer (200 mM NaH_2_PO_4_, 10 mM EDTA (pH 7.5)), 60 μl H_2_O, 10 μl (10 mM) NADPH and 10 μl (12 mM) DTNB, in multi-well plates. After addition of 10 μl GR, 10 μl of extract was added, after which DTNB reduction was monitored by measuring absorbance at 415 nm using the plate reader (PowerWave XS2, Bioteck). Without pre-treatment of extracts, the method measures total glutathione, that is, reduced glutathione (GSH) plus the oxidised glutathione (GSSG). Specific measurement of GSSG only was achieved by pre-treatment of extract aliquots with 2-vinylpyridine (VPD). To measure only the oxidized form GSSG, the same measurement was done after a pre-treatment for blocking the reduced form GSH present with 2-Vinyl-pyridine (1 μl). The final GSH and GSSG concentration were expressed in nmol.g^-1^ of biomass-wet weight, and the results from irradiated samples were normalized versus their representative non-irradiated controls and plotted as percentage.

### RNA extraction

Thirty ml of all irradiated and control *Arthrospira* cultures (at OD_750nm_ ~1), including three independent biological replicates per test condition, were centrifuged (20 min at 10 000 g, at 4°C) (Avanti J- 26XP; Beckman Coulter, Suarlée, Belgium) to collect the cells. Cell pellets were washed three times (12.000g, 5 min) with PBS (1X) pH 7.4. Then samples were flash frozen in liquid nitrogen and immediately stored at-80°C, until analysis (ca. 5 days). Frozen cells were suspended in 750 μl RNA Wiz solution (Ambion), and were disrupted mechanically by bead-beating using Zirconia Beads for 10 min, at room temperature, in the Vortex-Genie 2 apparatus (MoBio), using vortex adapters (13000-V1, Mo Bio). Next, the released RNA was separated from the cell debris by centrifugation (10 000 g, 10 min, 4°C) (Microcentrifuge 5418R, Eppendorf). RNA purification was performed using the RiboPure-Bacteria Kit (Ambion) following the manufacturer's instructions. The RiboPure-Bacteria Kit (Ambion) allowed the collection of larger RNAs (e.g. 16S and 23S ribosomal and messenger mRNA), but not the collection of small RNAs (e.g 5S ribosomal RNAs, tRNAs). Hence the flow-through form the purification with RiboPure-Bacteria Kit (Ambion) was carefully collected and further purified with a Direct-zol RNA MiniPrep 2050 (Zymo Research) to recover in addition the small RNAs. The final RNA products obtained from both purification processes were combined and used for preparing the RNA for microarray analysis. RNA samples (ca. 500 ng/μl, in a total volume of 70 μl) were then treated once with DNAse (Ambion TURBO DNA-free, Life Technologies Europe B.V, Ghent, Belgium) for 30 min at 37°C following the manufacturer's instructions to remove DNA. Absence of DNA was confirmed by PCR with DNA polymerase and universal 16S RNA gene primers. Finally, the RNA was concentrated using RNA Clean & Concentrator-25 (Zymosearch) to a concentration of ca. 250 ng/μl, in a total volume of 20 μl. This extraction method was found to yield sufficient quantity and superior quality of RNA, compared to the method based on heat shock lysis and hot Trizol (Invitrogen) extraction and Direct-zol RNA MiniPrep 2050 (Zymo Research) purification previously used [12]. RNA quantity and purity was assessed by spectrophotometric analysis using the NanoDrop ND-1000 Spectrophotometer (Thermo scientific). Samples had to meet the following minimum basic criteria: 260/280 > 2.0 and 260/230 > 1.8. The quality and integrity of RNA was assessed using the Bioanalyzer 2100 (Agilent) according to manufacturer's instructions. RNA selected for microarray had an RNA integrity number (RIN value) above 7.

### RNA analysis via microarrays

RNA extracts were analysed using our own designed *Arthrospira* HX12 microarray chips, having a 12x135k array format (Nimblegen). The '*Arthrospira* HX12' (Nimblegen, wI, USA) tiling array spans the full *Arthrospira* sp. PCC 8005 genome, with 135.367 probes ranging from 50 up to 72 nucleotides and an average length of 53 nucleotides, and an average spacing of 34 nucleotides between 2 different probes, which could be mapped back to 5854 CDS and 3141 intergenic regions (based on Genome version 5, privately available in the 'Arthroscope' database on the MaGe platform [18]). Microarray analysis was done at the Institute for Research in Biomedicine (IRBB) in Barcelona, Spain. cDNA library preparation and amplification were performed on 25 ng total RNA, obtained from three independent irradiated and control samples per test condition (n = 3), using the Complete Whole Transcriptome Amplification (WTA2) kit (Sigma-Aldrich), according to the instructions of the manufacturer, with 17 cycles of amplification, resulting in microgram quantities of cDNA. Labelling and hybridization of the cDNA onto the microarray were performed according to Roche-Nimblegen expression guide v5-p1. For each sample, 1 μg cDNA was labelled by Cy3 nonamer primers and Klenow polymerization. Subsequently, a hybridization mixture was prepared with 2 μg Cy3-labeled cDNA. Samples were hybridized to *Arthrospira* HX12 array (Nimblegen) for 18 hours at 42°C. The arrays were washed and scanned in a Roche-Nimblegen MS 200 scanner. Raw data files (Pair and XYS files) were obtained from images using DEVA software (Roche-Nimblegen) and provide by IRBB to SCK•CEN for further data analysis. Per irradiation dose and per time point, 3 microarrays of irradiated cultures and 3 microarrays of their equivalent non-irradiated cultures (n = 3) were analysed, resulting in 6 arrays per condition. Thus, for all 3 irradiation doses (800 Gy, 1600 Gy and 3200 Gy) and for all 3 time points after irradiation (0H, 2H and 5H) tested, this resulted in 36 arrays in total.

### Microarray data analysis

At SCK•CEN raw microarray data were pre-processed using the “Oligo” package (version 1.24) in BioConductor (version 2.12 / R version 3.0.1) as follows: i) background correction based on the Robust Multichip Average (RMA) convolution model [19], ii) quantile normalization to make expression values from different arrays more comparable [20], and iii) summarization of multiple probe intensities for each probe set to one expression value per gene using the median polish approach [19]. To test for differential expression between the different irradiated conditions and the reference conditions (no irradiation) the Bayesian adjusted t-statistics was used as implemented in the “LIMMA” package (version 3.16.4) [21]. P-values were corrected for multiple testing using the Benjamini and Hochberg’s method to control the false discovery rate (FDR) [22]. Transcripts were considered significantly differentially expressed when the corresponding adjusted p-value was lower than 0.05 and their Log_2_FC was equal or higher than 1 for up-regulated genes, and equal or lower than-1 for the down regulated ones, meaning fold changes (FC) of >2 or <0.5 respectively. The gene annotation was based on the expert annotation privately available in the 'Arthroscope' database on the MaGe platform [18], which is a manually curated annotation.

Genes having an absolute Log_2_ fold change higher than 1 and lower than-1 and a corrected p value <0.05, in each separate test condition, were displayed on a scatter plot. The total number, of differentially expressed genes after 800 Gy (2.4 min exposure) is respectively: 1485 at T0H, 181 at T2H and 203 at T5H. These numbers were higher after exposure to 1600 Gy (4.8 min exposure) resulting respectively in 2153 genes at T0H, 252 at T2H and 249 at T5H. Finally, the strongest expression and response was seen after exposure to the highest dose of 3200 Gy (9.6 min exposure), showing 2585 differentially expressed genes at T0H, 414 genes at 2H and 452 genes at T5H. In order to group genes that are co-expressed over the different time points and irradiation doses, the unsupervised soft clustering implementation Mfuzz was used (Mfuzz version 2.18.0; R-version 3.0.1) [23]. As input the fold changes of those genes having an absolute Log2 fold change higher than 1 and lower than-1 and a p-value corrected for multiple testing lower than 0.05 in either one of the 9 conditions, were selected, i.e. 1838 genes in total. A fuzzier value of 1.38 was applied, as calculated based on the method proposed by Schwammle and Jenssen [24]. The number of predefined clusters was arbitrarily set to 9. One gene can potentially belong to multiple clusters. Additionally, a Principal Component Analysis (PCA) plot was created, to visualize the similarity between two clusters by looking at the proximity of the cluster centres in the PCA plot on the one hand, and the overlap between the averages in expression profile between different clusters is visualized by the line width between the different cluster centres on the other hand.

Gene set enrichment analysis (GSEA) based on the Cluster of Orthologous Groups (COG) was obtained using the hypergeometric distribution. This analysis allowed deriving whether a gene cluster is containing a higher number of representatives of a specific COG then would be expected by chance. Visualization is performed in R (version 3.0.1) using the heatmap.2 command in the gplots package (version 3.4.1).

### Statistical analysis

The Graph Pad Prism software (version 5.00, GraphPad Software) was used, for preparing data graphs and for statistical analysis using One way ANOVA followed by "Dunnett Multiple Comparison Test" with confidence interval 95% (p < 0.05).

## Results

### Global gene expression dynamics

**Fig 1:**
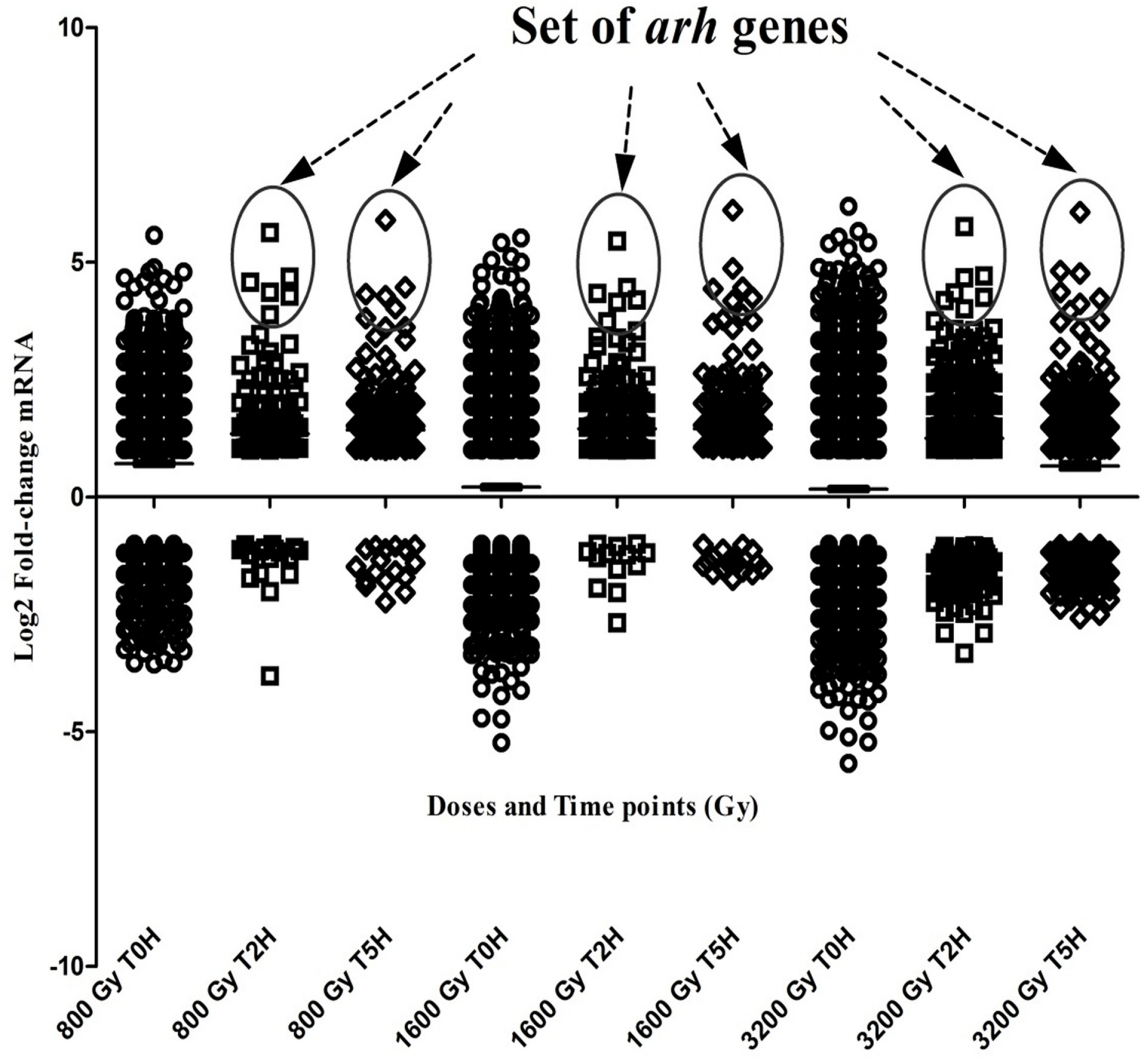
Scatter plot showing the differentially expressed genes of *Arthrospira* sp. PCC 8005 in response to gamma irradiation plotted accordingly to their change in mRNA concentration (Log_2_ fold change values), for 3 radiation doses (800, 1600 and 3200 Gy) and 3 time points after radiation (0 hours, 2 hours, 5 hours).

For all 3 different doses tested (800 Gy, 1600 Gy and 3200 Gy), the gene expression of *Arthrospira* in response to radiation exposure was monitored immediately (0H), two hours (2H) and five hours (5H) after irradiation. The transcription profiles showed an intense 'general emergency' response immediately after irradiation (0H), with a high number of differentially expressed genes (both up and down), while this number decreases significantly in following two hours and five hours recovery period. There was one specific set of genes, however, the *arh* genes, which remained highly expressed throughout the full recovery period. The higher the radiation dose, the more pronounced this global emergency response was induced (Fig 1).

Genes having an absolute Log2 fold change higher than 1 and a corrected p value <0.05, are displayed. The total number, of differentially expressed genes after 800 Gy are respectively: 1485 at T0H, 181 at T2H and 203 at T5H. These numbers were higher after exposure to 1600 Gy resulting respectively in 2153 genes at T0H, 252 at T2H and 249 at T5H. Finally, the strongest expression and response was seen after exposure to the highest dose of 3200 Gy, showing 2585 expressed genes at T0H, 414 genes at 2H and 452 genes at T5H. The circles highlight the top ranked expressed gene set, i.e. the *arh* genes, which were highly induced by *Arthrospira* sp. PCC 8005 for all irradiation doses and throughout the full recovery period after irradiation.

**Fig 2:**
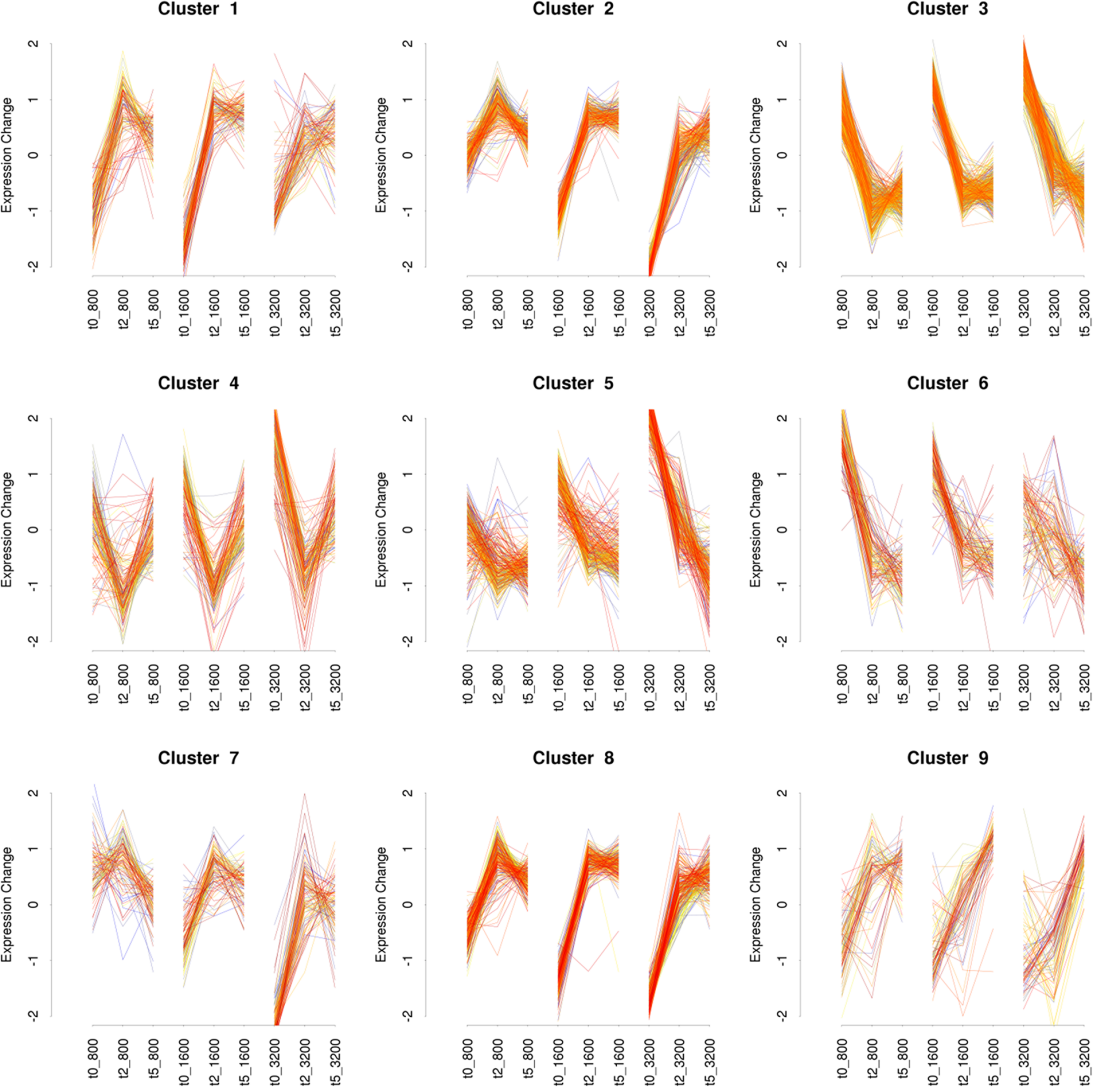
Dynamic changes in gene expression of *Arthrospira* sp. PCC 8005 in response to gamma irradiation, displayed in 9 clusters using the Mfuzz clustering software, according to their gene expression profile during recovery time (0 hours, 2 hours, 5 hours), for 3 radiation doses (800, 1600 and 3200 Gy).

To gain an overview of the major transcriptional response patterns, cluster analysis was performed on the differentially induced genes and the expression profiles of the different clusters over time (0H-2H-5H) were plotted, for each of the 3 doses (Fig 2).

Based on the PCA plot the clusters were assembled into two main different groups. The first group (E) contains clusters 3, 5, 6 and to a minor degree cluster 4, involved in the Early or Emergency response. In general, these four clusters group genes, which display the highest induced expression at time point T0H while their expression is fading out at later time points. The second group (R) includes clusters 1, 2, 7, 8 and to a lesser extent 9, involved in the Recovery response. In general, these five clusters group genes with lowered expression at T0H, that increases again significantly thereafter (T2H), with a clear impact of the radiation dose.

**Fig 3:**
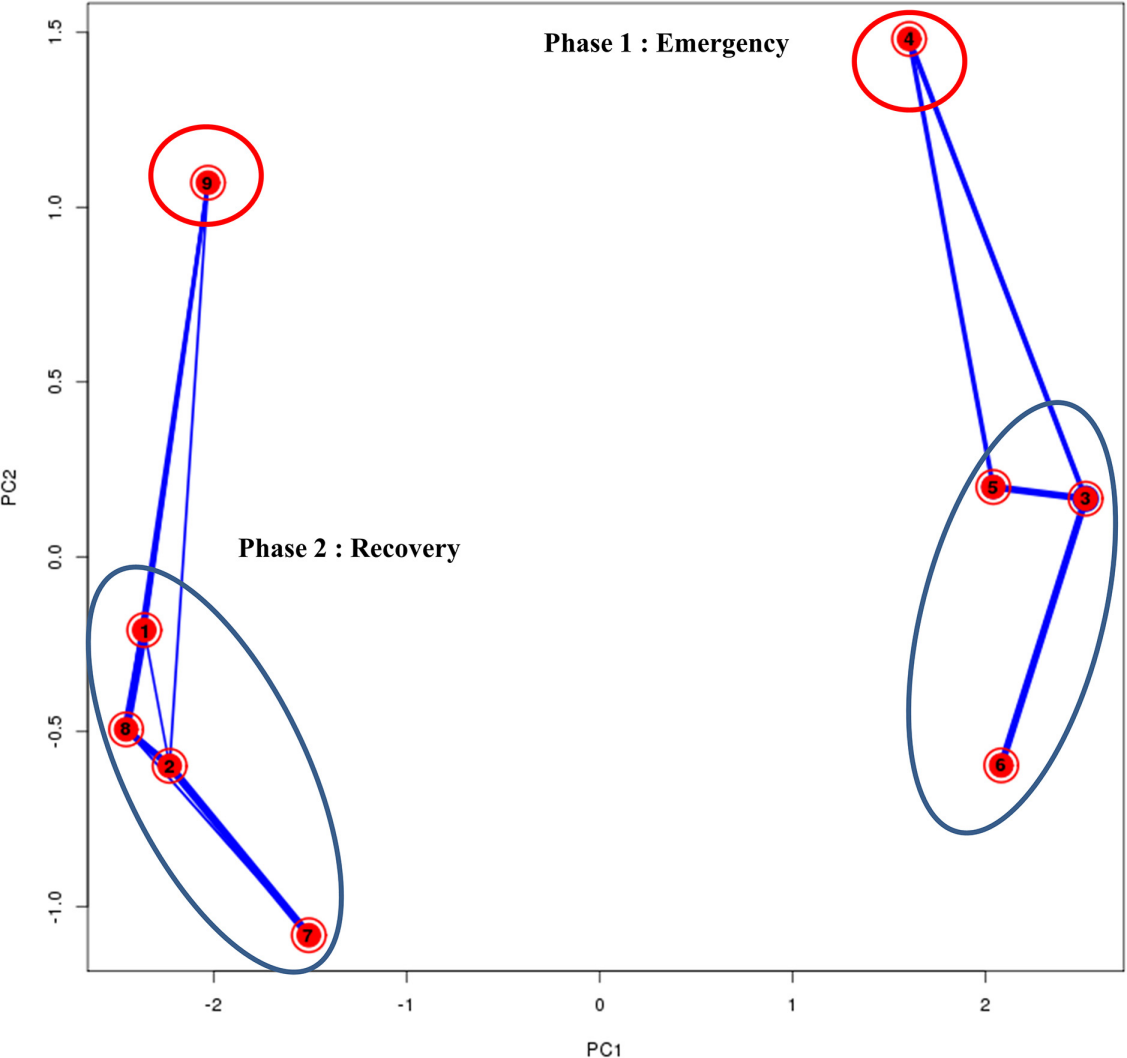
Principal Component Analysis (PCA) of the 9 cluster centres created using the Mfuzz clustering software for the gene expression of *Arthrospira* sp. PCC 8005 in response to gamma irradiation.

Thus the analysis of the global gene expression response revealed 2 'phases' in the dynamic transcriptional pattern: an Emergency response, activated immediately upon radiation exposure, followed by a Recovery response, in which genes that were silenced after radiation are reactivated later after irradiation (Fig 3).

This PCA biplot—plotting the first two principal components—gives a general idea on how the average expression patterns of the clusters are similar to each other. The closer the different cluster centres to each other, the more similar their average expression profiles are. Additionally, on top of the PCA plot, the overlap in genes between different clusters is visualized by lines with variable width between different clusters: the wider the line between two clusters, the higher the overlap. The graph generally shows a clustering in two groups, representing two distinct phases. Phase one—“Emergency response”: Clusters 3, 5 and 6 (blue ellipse) display the highest up-regulation at time point 0h and this effect fades out at later time points. To a lesser extent, also cluster 4 (red ellipse) can be categorized in the Phase1 response clusters, as genes belonging to this cluster display a significant high expression at 0H, and a strong repression after 2 hours and again a normal expression level after 5 hours. Phase 2 -“Recovery response”: Clusters 1, 2, 7, and 8 (blue ellipse) grouping genes with lowered expression at T0H that increased again during recovery; and in minor degree cluster 9 (red ellipse) showing significant increase in expression for all doses during recovery.

### Gene specific response patterns

#### Emergency (E)—Activation of protection, detoxification, and repair systems

Clusters 3 (460 genes), 5 (274 genes) and 6 (122 genes) contain genes which are strongly induced immediately after irradiation and which decreased in expression intensely after 5 hours. The transcriptomic profile of the cluster 5 showed the strongest induction for the highest dose of 3200 Gy, for cluster 6 this was the opposite with a stronger induction for the lowest dose of 800 Gy, and for cluster 3, induction levels were similar for all doses. These clusters contain genes involved in the activation of protection, detoxification, and repair mechanisms in *Arthrospira* during irradiation.

Immediately after irradiation, there was an up-regulation of a gene (*hyuA)*, involved in the synthesis of glutathione, an important intracellular metabolite for detoxification of ROS in plants, but rare in bacteria [25] (S2 Table). In line with glutathione synthesis, also a set of genes involved in proline synthesis (*proA1*, *putA*, *pep*) was induced, recognized as non-enzymatic antioxidant of microbes and plants to mitigate the adverse effects of ROS [26]. Radiation led also to the induction of (ARTHROv5_30341) gene potentially involved in the synthesis of the enzymatic antioxidant peroxiredoxine. Other genes from these clusters contributing to cell detoxification are involved in metal homeostasis. The results showed an induced expression of inner membrane transporters responsible for the import of various redox metals which are cofactors for many enzymes, such as iron (*feoA*, *feoB*, *fur*), copper (*cutA*), magnesium (*corA*, *mtgC*), cobalt (*cbiQ1*, *cbiQ2*), zinc (*znu*A), and potassium (ARTHROv5_61130) (S2 Table). But there was also induced expression of genes responsible for selective export of metals such as copper (*copA1*) (S2 Table). The induced expression of genes coding for outer membrane *ompA*-like porins and permeases may have helped for the efficient diffusion of small and hydrophilic solutes over the outer membrane (S2 Table).

Exposure to radiation also strongly induced the transcription of genes involved the clean-up of damaged proteins, peptides and amino acids, such as proteinases and peptidases (*clpB2*, *patG*) that remove dysfunctional proteins, and some chaperones (*hspA*, *dnaK1*, *dnaK2*, *dnaJ*, *cbpA*). In addition, the expression of the *sufRS* genes was induced (S2 Table). The *sufRS* genes are involved in the "sulphur assimilation pathway" (SUF), which can be used an alternative pathway for the "iron-sulphur cluster pathway" (ISC) (which involves the *iscA1* gene, of which the expression was reduced) under oxidative stress, and which can contribute to the FeS assembly. Several genes involved in the production of the amino acid glutamate under nitrogen starvation, including the genes for aspartate aminotransferase (*aat1*), amino acid transport (*lysE*) and degradation of urea (*ureABCDF)*, were significantly induced in a dose dependent manner (S2 Table). Surprisingly, also genes with a predicted function in nitrogen fixation (*nifU*, *devA*) showed induced expression (S2 Table), although *Athrospira* is described as non-heterocyst forming and non-nitrogen fixating [27].

The cells strongly induced several genes involved in the clean-up of nucleotides released from DNA damage repair. This includes the ADP-ribose pyrophosphatase (*nudE*), and the NUDIX hydrolase (*mutT*) (S2 Table). The DNA repair system of *Arthrospira* involved the activation of nucleotide excision repair (*uvrBCD)*, mismatch repair (*mutT)*, gene *ruvB* involved in resolving holiday junctions, and other helicases and DNA repair genes (*dnaG*, *mod* and *recJ)* (S2 Table). In addition, several systems for DNA modification and protection, such as the endonuclease genes (*pvuIIR*) and the Enzymatic Restriction Modification genes (*hsdR*), were highly induced (S2 Table). In parallel, there was a high induction of phage-like genomic islands (*fax* genes), the phage immunity system (CRISPR) (*cas* genes) and a toxin/antitoxin system (S2 Table). The synthesis of nucleic acids and nucleotides was rerouted via the pentose phosphate pathway, via the expression of the ribokinase gene (*rbsK*).

The carbon metabolism seemed to be oriented towards the degradation of glycogen (*sigE)*, the synthesis of intracellular C-storage molecules such as trehalose (ARTHROv5_41060) and polyhydroxyalkanoates (*phaC*) (S2 Table). Cells induced also genes for sugar (*ugpB*) and polyamine (*potBC)* import (S2 Table). The later may participate in import of spermidine, well known as a vital compound for cell survival for plants and eukaryotes [28]. Interestingly, the cluster also includes a set of genes involved in cobalamin biosynthesis (*cbiM2*, *cbiQ2*, *cobW*), which is a precursor of vitamin B12 that is involved in DNA synthesis and regulation, but also amino acid metabolism and fatty acid metabolism. The activation of lipase and esterase lipase genes suggested lipid degradation.

**Fig 4:**
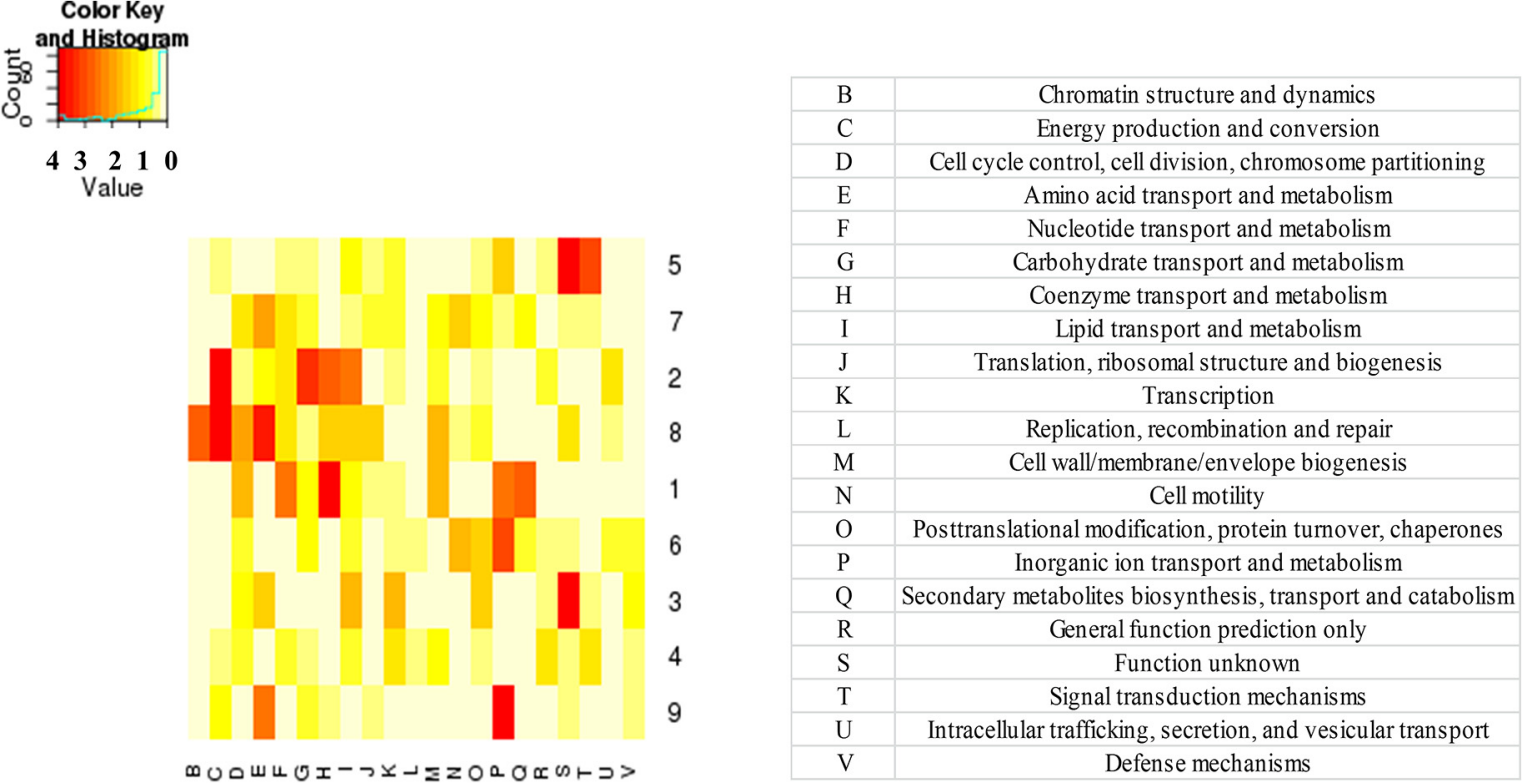
Gene Set Enrichment Analysis (GSEA) in the clusters of differentially expressed genes of *Arthrospira* sp. PCC 8005 in response to gamma irradiation based on the Clusters of Orthologs Groups (COG) functional categories. This plot visualises whether a certain gene cluster (1–9, on the vertical axis) is containing a higher number of representatives of a specific COG (21 different COGs, on the horizontal axis) then would be expected by chance. The COG functional category is shown in the vertical direction, the clusters of differentially expressed genes in the horizontal direction. The colour code is according to the ^10^log value of the corresponding p-value of the GSEA analysis: a p-value of smaller than 1.10^–4^ (^10^log-value of 4) results in a colour code red, a p-value of 1 (^10^log value 0) results in colour code white.

Cluster 5 was significantly enriched with genes belonging to the category related to the signal transduction system (COG—T) (Fig 4).

Genes coding for response regulatory proteins with photosensor domains (GAF, PAS), as well as the genes for synthesis of the secondary messenger cyclic diguanylate (c-di-GMP) and the associated signal transduction and regulatory systems (i.e. histidine kinases and transcriptional regulators) were induced. In addition, the induction of the photo-sensor Cry DASH (*cry* gene) was observed, well known in cyanobacteria as photosensor to control UV-A induced phototaxis [29].

#### Recovery (R)—Restart of photosynthesis, carbon fixation, and nitrogen assimilation systems

Clusters 1, 2, 7, 8 and 9 responded in the opposite way compared with clusters 3, 5, and 6 mentioned above. They group those genes with lowered expression upon irradiation that increased again gradually during the recovery period. Clusters 2 and 8, were very similar in expression profiles, and showed the strongest response for the highest dose (Fig 2). Genes from Cluster 1 responded in a similar way as Cluster 2 and 8 for 800 Gy and 1600 Gy, but had a less pronounced response to 3200 Gy.

Clusters 1, 2 and 8 were significantly enriched with genes belonging to the COG category of Energy production and conversion (C) and/or Coenzyme transport and metabolism (H) (Fig 4). The genes of cluster 7 were also strongly repressed after irradiation, mainly at the highest dose (3200 Gy), but slightly induced after two hours recovery period, and back to original expression after five hours (Fig 2). Cluster 7 was significantly enriched with genes involved in the category of amino acid biosynthesis and transport (E) (Fig 4). Cluster 9 grouped genes which were slightly repressed after irradiation and induced during the recovery period, but required up to 5 hours to reach normal expression levels. Cluster 9 was significantly enriched with genes belonging to the category of Inorganic ion transport and metabolism (P) (Fig 4).

Overall, the genes grouped in these clusters were mainly involved in photosynthesis, carbon fixation and nitrogen assimilation. These clusters contain genes involved in (i) the synthesis of structural proteins of the phycobilisome and their linker polypeptides (*apcAB*, *apcF*, *apcC*, *cpcC1*, *cpcE*), the PSII (*psbBNO)* and PSI (*ycf4*, *psaE*) complexes, (ii) the biosynthesis of pigments and cofactors such as porphyrin and haem (*hemCE*, *hemG*, *hemL*, *hemN1*, *cobA*), and chloropyll (*bchD*, *chlP*, *chlGH*), and (iii) the electron transport chain (*ndh*, *coxABC*, *cydB*). Also genes involved ATP production (*atpF*, *atpG2*, *atpH*) were silenced immediately after irradiation (S3 Table). The expression of the hydrogenase genes (*hypA1*, *hypB1*) was not really reduced upon radiation, but strongly induced during recovery (S4 Table).

The energy collected from photosynthesis in the form of NADPH and ATP is normally used for CO_2_ fixation and synthesis of carbohydrate molecules via the Calvin-Benson-Bassham (CBB) cycle and also the genes for this process (*cbbR*, *pgI*, *pgk*, *xfp*) were strongly reduced in expression immediately after irradiation (S3 Table). In line with the reduced carbon capture, also genes involved the Krebs cycle (also called tricarboxylic acid TCA cycle) providing precursors of certain amino acids (*sdhA*, *sucD*, *sucC*), and in biosynthesis of lipids such as fatty acids (*fabF2*, *fabG1*, *fabZ*, *fabG2*, *fabH*, *des)* showed a reduced expression (S3 Table). Also the expression of genes for biosynthesis of intracellular solute glucosylglycerol with a role in salt tolerance (*stpA*, *ggpS*) was significantly reduced (S3 Table). Cluster 1 and 8 contain in addition genes involved in cell envelope synthesis (*mrdA1*, *murABCDE)*, and motility (*pilT1*), which were all also reduced in expression (S3 Table).

Carbon metabolism is tightly correlated with nitrogen metabolism, and many genes related to nitrogen assimilation and metabolism, were immediately repressed (*glnB*, *ntcA)* upon irradiation. Many genes of cluster 9 are involved in transport and assimilation of nitrogen sources, including for example genes for nitrate and nitrite uptake (*nrtABDC*, *nrtP*, *narB)*, cyanate uptake *(cynBD)*, and amino acid uptake, transport and biosynthesis (*livGMHJ*, *aapJ*, *aapP*, *aapQ*, *iaaA*, *argG*, *argH*, *argJ*) (S3 Table). Also the genes for the assimilation of ammonium in glutamate amino acids via the glutamine synthetase pathway (*glnA*, *glsF*), the uptake and degradation of organic N-polymers, such as nitrile (*nthA1*, *nthB2*), the hydrolysis of the polyamine agmatine to putrescine and urea (*speB*), and the biosynthesis of the extracellular cyclic peptide patellamide A (*patABC)* were immediately shut down (S3 Table). In contrast, the nitrogen metabolism seemed to be rerouted to polyamide uptake (*potBC)*, urea hydrolyses (*ureABCDF*) leading to ammonia production, and ammonium assimilation and glutamate amino acids production via the aspartate aminotransferase pathway (*aat1*), which were highly induced upon irradiation, as was mentioned above (S2 Table).

Cluster 1 and 8 contain also the *arh* genes, a gene set coding for proteins with unknown function which we recently discovered to be specifically produced in *Arhtrospira* sp. PCC 8005 in response to high doses of gamma rays (3200 & 5000 Gy). This gene cluster, composed of five genes named *arhABDEF*, showed here also a very high and dose-dependent transcription in response to lower doses of ionising radiation (S5 Table). This gene cluster was among the top ranked differentially expressed genes identified immediately after irradiation, and remained highly expressed during the recovery (Fig 1). Next to the gene involved in the biosynthesis and regeneration of glutathione (*gshB)*, of which the expression was clearly induced during recovery, the only gene set displaying such strong expression profile (S4 Table).

### Glutathione measurement

**Fig 5:**
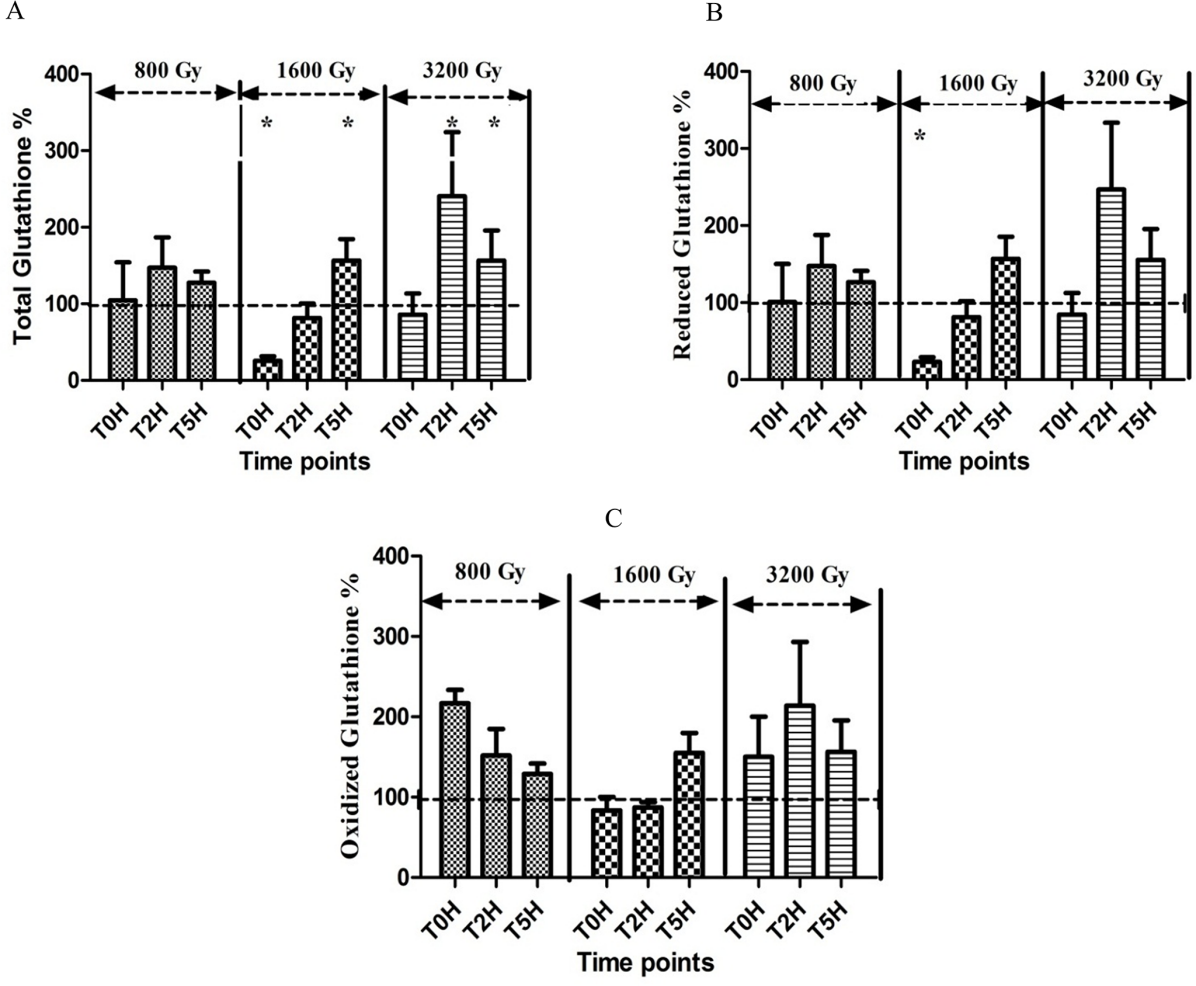
Intracellular glutathione concentrations of *Arthospira* sp. PCC 8005 after irradiation. A, B and C show the normalised intracellular concentrations of respectively total glutathione (GSH+GSSG), reduced glutathione (GSH) and oxidized glutathione (GSSG) immediately (T0H), 2 hours (T2H) and 5 hours (T5H) after irradiation. The data obtained for the irradiated samples were normalized against and are shown as percentage of their representative non-irradiated control (which was put at 100%). Data represent the mean of three independent biological replicates, and error bars present the standard error of the mean (SEM). The statistical analysis was calculated on the raw data, before normalisation to percentages. One asterisk indicates a value which is significantly (p<0.05) different from the value of the non-irradiated control.

The intracellular glutathione concentration of *Arthrospira* sp. PCC8005 was assessed immediately after and during the five hour recovery period following irradiation. While no significant change was noted for cells exposed to the lowest dose (800 Gy), there was a significant increase of total glutathione concentration in the cells exposed to higher doses (1600 and 3200 Gy), two hours and five hours post irradiation (Fig 5). This is consistent with the expression profile of the genes involved in the biosynthesis and regeneration of glutathione (*hyuA*, *gshB)*, described above. The extra glutathione was mainly present in the reduced form, as the level of the oxidized glutathione did not change significantly.

### Photosynthesis efficiency and pigments

**Fig 6:**
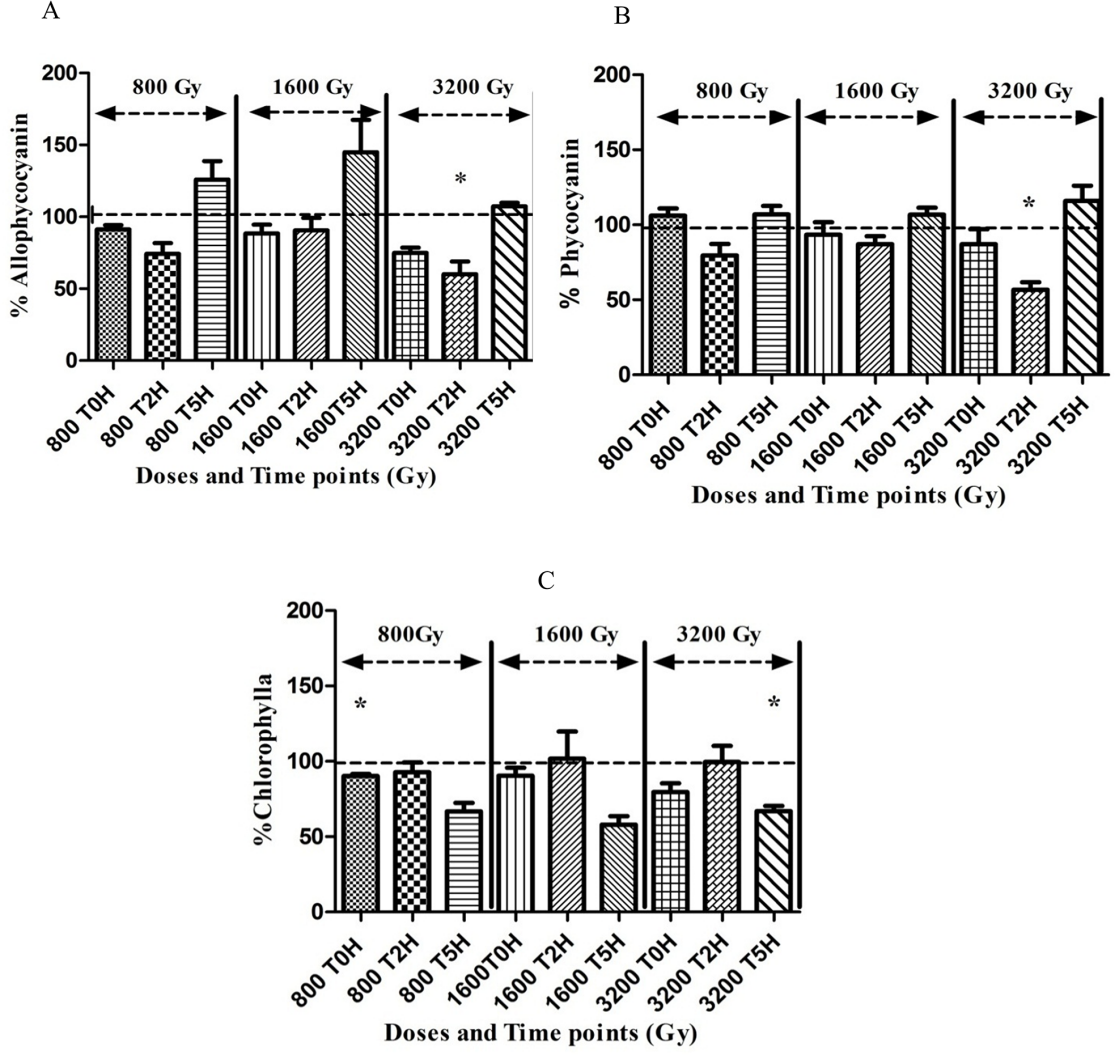
Pigment content of *Arthrospira* sp. PCC 8005 after irradiation. The data obtained for the irradiated samples were normalized against and are shown as percentage of their representative non-irradiated control (which was put at 100%). Allophycocyanin content, Phycocyanin content, and Chlorophyll A content were measured at T0H, T2H and T5H respectively. Data represent the mean of three independent biological replicates, and error bars present the standard error of the mean (SEM). The statistical analysis was calculated on raw data, before normalisation to percentages. One asterisk indicates a value which is significantly differing (p<0.05) from the non-irradiated control.

**Fig 7:**
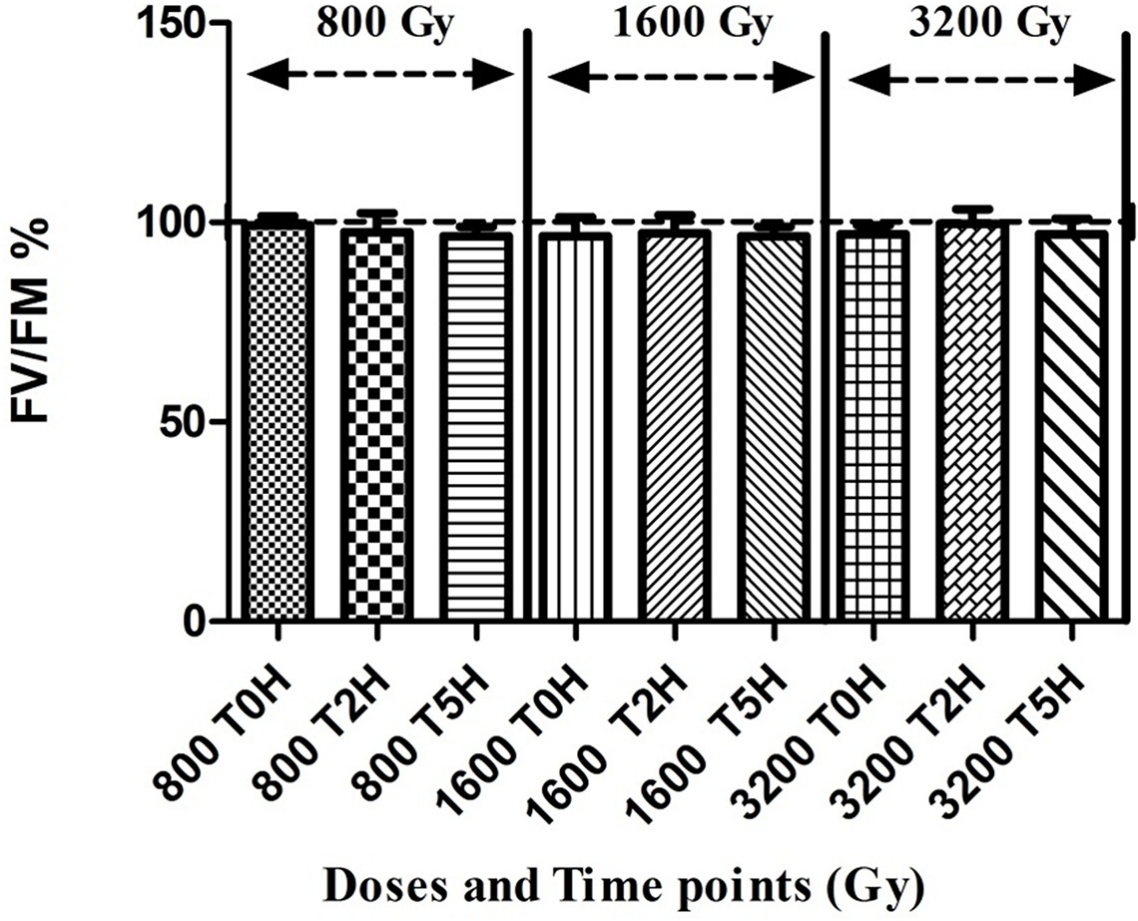
Photosynthetic capacity of *Arthrospira* sp. PCC 8005 after gamma irradiation. The data obtained for the irradiated samples were normalized against and are shown as percentage of their representative non-irradiated control (which was put at 100%). Data represent the mean of three independent biological replicates (n = 3) and error bars display the standard error of the mean (SEM). The statistical analysis was calculated on raw data, before normalisation to percentages.

For photosynthetic growth, *Arthrospira* requires photosynthetic pigments and an active photosystem. The analysis of the photosynthetic pigments, showed very little change in the overall pigment content in the cells after irradiation. Nevertheless, there seemed to be a trend showing that phycocyanin and allophycocyanin content slightly decreased in the first 2 hours after radiation, but was restored to a normal level during recovery 5 hours later (Fig 6). For chlorophyll this slight decrease was only observed 5 hours after irradiation. This trend was more pronounced for the higher dose of 3200 Gy. The functionality of the photosystem was assessed by measuring the PSII quantum yield via fluorescence. The results showed no adverse effect of irradiation on the photosynthetic potential of the cells, even after the highest dose 3200 Gy (Fig 7). The PSII quantum yield of irradiated cells was stable immediately, 2 hours and 5 hours after irradiation and similar to non-irradiated healthy *Arthrospira* cells displaying a F_V_/F_M_ yield of ca. 0.6 [15].

### Survival, recovery and proliferation

The *Arthrospira* sp. PCC 8005 cultures were indeed able to fully recover normal photosynthetic growth after exposure to all 3 different doses tested, i.e. 800 Gy, 1600 Gy and 3200 Gy (S1 Fig). Growth curves showed total recovery of photosynthetic growth after all exposures, although with significant delay at 1600 Gy and 3200 Gy (up to 8 days for 3200 Gy) (S1 Table). Non-irradiated samples and samples irradiated with 800 Gy obtained a maximum specific growth rate between day 1 and 3 respectively. For cells exposed to 1600 Gy, maximum growth rate was only seen between day 3 and 6, and cell exposed to 3200 Gy needed 14–17 days to reach maximum growth rate respectively (S1 Table). The exposure of *Arthrospira* sp. PCC 8005 in this set-up to 10 000 Gy of ^60^Co gamma rays was lethal, this dose killed all the cells, after which no recovery was resumed (S1 Fig).

## Discussion

*Arthrospira* sp. PCC 8009 is highly resistant to gamma radiation [12]. A dose of 10 000 Gy is lethal, but cells can maintain photosynthetic capacity and can recover photosynthetic growth after exposure, for all doses tested up to 3200 Gy. The radiation had little effect on the morphology, pigmentation, or photosynthetic capacity of the cells. Despite these little physiological changes, there was however, a strong gene-expression reprogramming in the cells exposed to radiation. The higher radiation dose, the more pronounced the transcriptional and physiological response of the cells, and the longer the delay in restart of active photosynthetic growth after irradiation.

The dynamic transcriptomic response showed two waves: an early 'emergency-type' response that occurred immediately upon irradiation and a recovery response during the two and five hours after irradiation. The acute exposure to high doses of radiation, induced a clear 'shock' in the cells, which showed the largest changes in transcription immediately after irradiation. *Arthrospira* sp. PCC 8005 cells, immediately expressed and supressed a high number of genes, even without direct correlation to physiological changes. During early response, transcriptome analysis showed a significant repression of genes involved in photosynthesis, carbon, and nitrogen assimilation; and the higher the radiation dose, the longer the reduced expression was maintained. Although overall pigment content and the photosynthesis capacity (PSII quantum yield) measurements did not reveal a drastic irradiation effect, the active renewal of the proteins involved in photosynthesis was temporarily put-on hold during the irradiation and only switched back-on gradually after irradiation during the recovery. The short irradiation time (minutes), the long half-life of the proteins involved, and the lack of an active phycobilisome degradation via NblA enzyme (whose gene expression was not altered) [30], may explain why photosynthesis measurement revealed intact and functional photosystem even after exposure to the highest dose 3200 Gy.

The reduced transcription for photosynthesis in response to radiation was combined with a reduced transcription of the photosynthetic energy production pathway, i.e. the ATP production systems. Our results did show, however, the active transcription of *hypA1* and *hypB1* required for the nickel insertion step of [NiFe]-hydrogenase maturation. This enzyme is a bidirectional hydrogenase, a Ni-Fe metalloenzyme, for reversible reduction of protons (H^+^) to di-hydrogen (H_2_) [31, 32]. The physiological role of this enzyme, however, has been a matter of speculation and is still unclear. Nevertheless it has been proposed that this enzyme functions as a valve for low potential electrons generated during the light reaction of photosynthesis, thus preventing slowing down the electron transport chain under stress conditions [33]. This enzyme is known to stimulate metabolic processes in darkness, with reduced supply of bicarbonate, nitrate or sulphate and other environmental stresses [34]. High rates of hydrogen production have been obtained in *Arthrospira sp* PCC 8005 cells adapted to nitrogen and sulphur deprived medium supplemented with iron and beta-mercapto-ethanol [35].

With the reduced transcription for photosynthesis and energy production, cells reprogrammed also the expression of genes for carbon metabolism during irradiation. For instance, several genes involved in de Calvin-Benson-Bassham-cycle (CBB-cycle) that fixes carbon dioxide into glyceraldehyde-3-phosphate (G-3-P) and finally hexose sugars such as glucose, and the Krebs cycle (also known as the tricarboxylic acid (TCA) or the citric acid cycle) providing the precursors for amino acids biosynthesis, were significantly repressed in early response. This is consistent with reported findings that showed the repression of TCA pathway cycle immediately after irradiation of *Deinocccus radiodurans* as well as during the first hours of the recovery period [36, 37]. The carbon metabolism gene expression profile showed that irradiation caused a re-routing of the metabolic flux to glycolysis and the pentose phosphate pathway, in favour for NADPH and pentoses generation [38]. NADPH and pentose molecules are essentially required to provide the cell with ribose-5-phosphate (R5P) for the synthesis of the nucleotides and nucleic acids, and may act as cofactor for glutathione and thioredoxin reductases [39]. In addition, *Arthrospira* sp. PCC 8005 cells rerouted their carbon metabolism to synthesis of storage molecules in the form of trehalose and polyhydroxyalkanoate (PHA). Several reports suggest the contribution of trehalose in response to high salt stress [8], and in desiccation tolerance by protecting the cells and proteins from oxygen radicals [40]. Recently Webb and co-worker suggested a possible role of trehalose as an efficient protectant of protein activity (enzymes) against irradiation, either alone or in combination with Mn^2+^. The addition of trehalose resulted in a significant increase in enzyme protection, up to 6 000 Gy (given at a dose rate 3 200 Gy hr^-1^) of ^60^Co gamma rays [41]. Trehalose can also stabilize membranes and maintain their integrity and fluidity [42]. The intracellular store of carbon in the form of polyhydroxyalkanoates (PHA) enables most cyanobacteria to survive stress conditions, such as micronutrient starvation (e.g. nitrogen) and to recover rapidly [43]. It has been reported that during desiccation *Micrococcus vaginatus* induces the transcription level of its PHA biosynthesis genes [44].

Furthermore, our findings showed an altered nitrogen uptake, assimilation and metabolism. Nitrogen is, however, an essential component for synthesis of amino acids for structural proteins and enzymes, nucleotides for DNA and RNA, and amino sugars for lipopolysaccharide and peptidoglycan in the cell envelope [45]. In general, genes for nitrogen uptake and assimilation were strongly reduced in expression after irradiation. This includes nitrate, nitrite, cyanate, and amino-acid uptake, and nitrile hydrolysis. It is well known that cyanophycin (L-Arginine and L-Aspartic acid) is thought to represent a dynamic reservoir of nitrogen accumulated in both non-nitrogen fixing and filamentous or unicellular N_2_-fixers, responding to the N regime [46]. Under nitrogen limiting conditions cyanophycin is catabolized as an internal nitrogen source, balancing the nitrogen deficiency. However, our findings showed no differential expression of the cyanophycinase genes (*cphA*, *cphB)* responsible for this process in response to irradiation, confirming that the cells did not seem to experience N-limitation. At the contrary, the nitrogen metabolism seemed to be also adjusted to deal with high ammonium concentrations, possibly released from radiation damaged proteins, nucleotides and amino-sugars, which are known to be highly toxic to the cell. The ammonium (NH_4_^+^) produced from degradation of amino-acids, is usually quickly neutralised via urea synthesis [47]. There was after irradiation indeed also an increased transcription of urease genes in *Arthrospira* for the decomposition of urea to CO_2_ and ammonia (NH_3_), which provides ammonia for glutamate and glutathione production. There was also a clear deactivation of genes involved in polyamine degradation, *i*.*e*. agmatine degradation to putrescine and urea, and a clear transcriptional activation of polyamines import transporters (e.g spermidine and putrescine). The agmatine is a competitive inhibitor of polyamine transport. This is possibly a mechanism to prevent even more intracellular urea and ammonium production, and at the same time to maintain or increase the intracellular polyamine content. Polyamines are a group of nitrogen (amine) containing compounds that received recently considerable attention owing to its possible role in abiotic stress resistance [48]. Studies on polyamine transport in cyanobacteria have been scarce, but the implication of polyamine transport to protect *Synechosystis* against salt stress has been reported [49].

The regulation of the cellular response to C and N nutrient stress is typically under the control of the metabolic signal 2-oxoglutarate (produced in the TCA cycle), which interacts with the P_II_ protein (encoded by the *glnB* gene), a sensor and transcriptional regulator, which in its turn induces the expression of genes involved in controlling carbon and nitrogen metabolism. It has been reported that P_II_ protein regulates for example the transcription of the global transcriptional regulators NtcA, which in turn controls the expression of *nblA* involved in phycobilisome degradation [50, 51]. In our previous study, the *nblA* gene was significantly induced after exposure of *Arthrospira* sp. PCC 8005 to high acute doses of gamma rays (5000 Gy) [12], but not significant at the lower doses tested here (800 Gy, 1600 Gy, 3200 Gy). The data of this study showed actually the repression of the *glnB* and *ntcA* genes after irradiation, indicating that carbon and nitrogen metabolism was shut down.

The growth of cyanobacteria cells depends on a tight balance in Carbon/Nitrogen ratio. Overall, this temporarily reduced expression of proteins involved in photosynthesis, carbon and nitrogen assimilation immediately after radiation, are likely responsible for the observed delay in growth and the lower maximum specific growth rate achieved in cultures after exposure to 1600 and 3200 Gy. Similarly, it was shown that the growth rate of *Synechocystis sp*. PCC 6803 was retarded after 1 W m^−2^ UV-B radiation due to the reduction in amino-acid biosynthesis [52].

Furthermore, our results suggest that while photosynthesis and growth was temporarily put on hold, *Arthrospira* sp. PCC 8005 counteracts the cell damage caused by gamma rays via an increased expression of a wide range of protection, detoxification, and repair systems. The classical enzymatic way for anti-oxidative defence relies mainly on catalase and superoxide dismutase (SOD), well reported to be highly induced and essential for survival of cyanobacteria exposed to various stresses [53]. However, the catalase gene is absent in the *Arthrospira* sp. PCC 8005 genome and the single SOD gene was not induced in *Arthrospira* sp. PCC 8005 after exposure to gamma radiation, not in our previous studies and not in this study. The lack of significant expression of the SOD gene in response to irradiation might be due to its continuous high expression during normal conditions (i.e. in absence of irradiation), but would need to be confirmed, or could mean that other (non-enzymatic) antioxidant systems are used. *Arthrospira* indeed seemed to rely on different ROS detoxification and redox balancing methods to resist ionizing radiation. *Arthrospira* used essentially thiol-based anti-oxidant enzymes and molecules, such as peroxiredoxine and glutathione. Transcription in *Arthrospira* showed the activation of different genes involved in biosynthesis and regeneration of glutathione: the enzyme 5-oxoprolinase (*hyuA*) catalyses the generation of glutamate from 5-oxoproline, the enzyme Glutamate Cysteine Ligase (GCL) converts glutamate to gamma-glutamylcystein, and the enzyme glutathione synthetase (*gshB*) converts the precursor gamma-glutamylcystein to glutathione. Proline is the main precursor for glutathione synthesis and our findings suggest also a synthesis of proline in response to radiation. In addition, proline itself is also considered as potent antioxidant; it has been proposed that free proline can act as hydroxyl and singlet oxygen scavenger, inhibitor of lipid peroxidation and osmoprotectant [54, 55]. Our results also showed the up-regulation of the *putA* gene contributing in glutamate synthesis from proline. PutA is a flavoprotein with mutually exclusive functions as membrane-associated enzyme and a transcriptional repressor (58). The switch between the two activities is due to conformational changes triggered by proline binding. In the presence of proline, PutA is associated with the cytoplasmic membrane and acts a bifunctional enzyme catalyzing both reactions of the proline degradation pathway: the oxidation of proline to pyrroline-5-carboxylate (P5C) by proline dehydrogenase and subsequent oxidation to glutamate by pyrroline-5-carboxylate (P5C) dehydrogenase [56]. In the absence of proline, PutA is cytoplasmic and functions as a transcriptional repressor of the *put* regulon (58).

Glutamate synthesis was also induced via asparte-aminotransferase catalysing the formation of glutamate from aspartate and 2-oxoglutarate (also called α-ketoglutarate) from the TCA cycle. Glutamate can also be synthesised from 2-oxoglutarate, via the GS-GOGAT pathway or the dehydrogenase (GLDH) pathway [57]. The GS-GOGAT pathway (also called glutamine synthetase (GS) or glutamate synthase (GOGAT) pathway) was immediately shut-down after irradiation; while the glutamate dehydrogenase (GLDH) pathway was induced. Both pathways provide glutamate synthesis from NH_3_ and 2-oxoglutarate, but normally the GS-GOGAT pathway is used at low ammonium concentrations and when the cell is not under energy limitation (i.e. with sufficient reduced ferrodoxin or NAD(P)H), while the GLDH pathway is used when the cell is limited for energy and carbon but ammonium and phosphate are present in excess [58]. It is known that high ammonia availability (as discussed above) leads to repression and deactivation of GS-GOGAT and induction of GLDH [59]. *Synechocystis sp*. strain PCC 6803, for example, utilizes the GS-GOGAT pathway as the primary pathway of ammonia assimilation, but the presence of GLDH appears to offer a selective advantage for the cyanobacterium under non-exponential growth conditions [60]. The actual production of glutathione, which increased significantly during recovery, was confirmed via glutathione metabolite concentration analysis. Glutathione molecules are well known to play an important anti-oxidative role in the defence system of plants [61], and thus seem to provide the same benefits for cyanobacteria. Moreover, also the radiation resistant bacterium *Deinococcus radiodurans* contains a thiol-based antioxidant, called bacillithiol (BSH), which is considered as a substitute for glutathione [37, 62, 63]. Nevertheless, the role of BSH in radiation resistance remains to be confirmed, and would require the production and analysis of BSH-deficient mutants.

**Fig 8:**
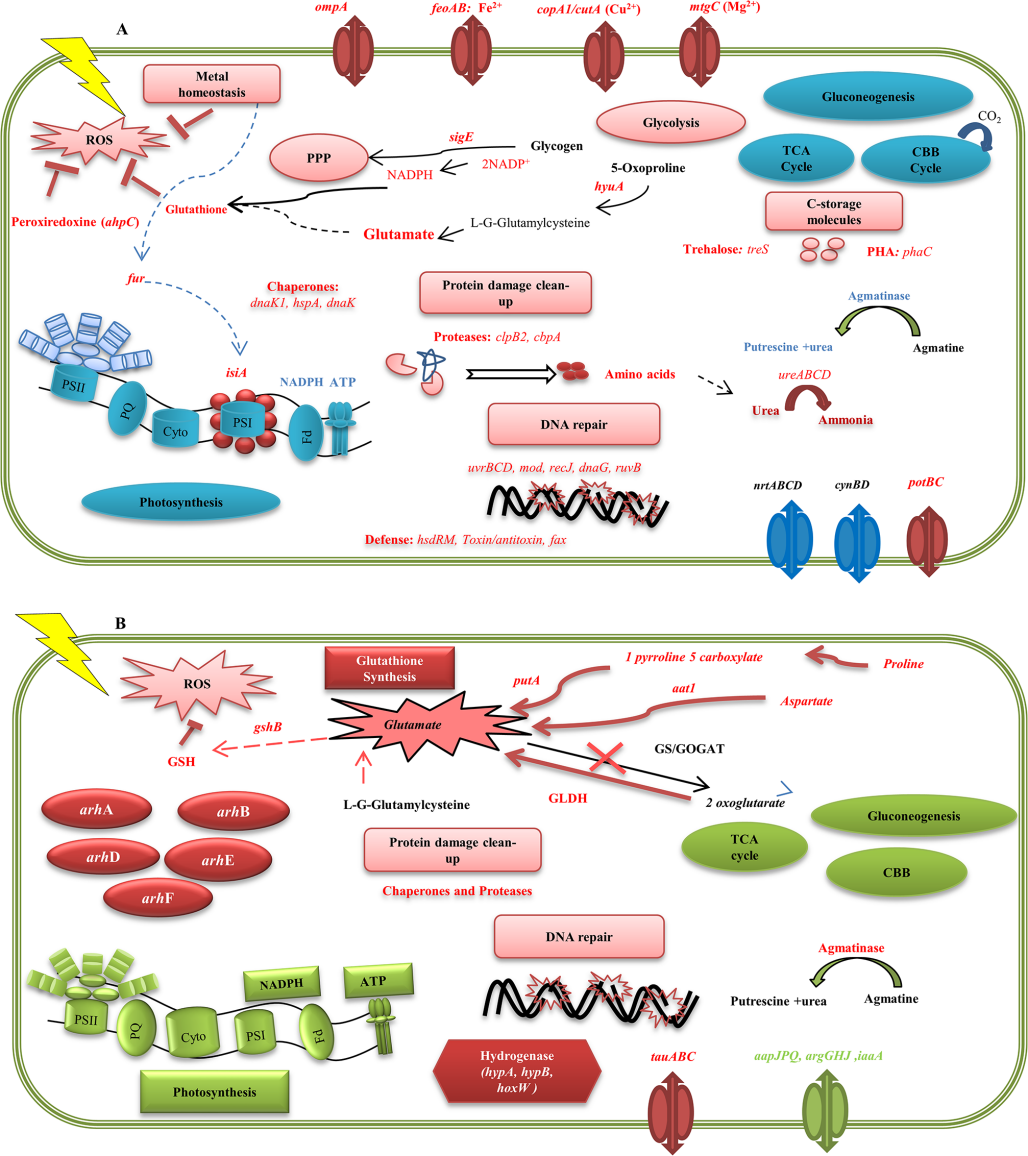
General overview of the main transcriptional response events of *Arthrospira* sp. PCC 8005 after exposure to ^60^Co gamma rays. Schemes represent a global gene expression response (A) immediately after irradiation; (B) after 2H and 5H of recovery period. Blue colour, stand for down-regulated genes. Red colour stand for up-regulated genes, Green colour stand for restored expression of the initial silenced genes. (A) The largest changes in transcription occurred upon irradiation, as part of a kind of an “Emergency Response”. Cells displayed a **reduced transcription** for photosynthesis and energy production (PSII, PSI, ATP), and for carbon and nitrogen metabolism during irradiation. The CO_2_ fixation via the Calvin-Benson-Bassham cycle (CBB), glycogen biosynthesis (gluconeogenesis) and the tricarboxylic acid cycle (TCA) were repressed. The transcription of the SigE regulator acting as nitrogen-dependent activator for catabolic genes towards glycogen degradation (glycolysis) was induced. Also a re-routing of the metabolic flux to glycolysis and the pentose phosphate pathway (PPP) was seen. A synthesis of carbon storage molecules (PHA) and compatible solutes (trehalose) was seen. The expression of polyamine import (*potBC)*, well known as a group of nitrogen-containing C-compounds which help in cell survival during stress, was recorded. The import of nitrate or cyanate as N-sources was repressed (*nrtABCD*, *cynBD*). In parallel also the metabolism of agmatine, a known competitive inhibitor of polyamine transport, was repressed. The cellular protection, detoxification, and repair were **enhanced** immediately after irradiation. In an effort to maintain the intracellular redox balance while provide sufficient metal-cofactors for enzymes, selective metal export (*copA)* and import (*feoAB*, *cutA*, *corA*, *mtgC*, *cbiQ1*, *cbiQ2*, *znuA*) was induced. There was upregulation of isiA gene encoding the CP43’ protein, which is an auxiliary antenna complex, to compensate for the loss of phycobilisomes. This protein may also serve as a chlorophyll storage molecule contributing to the reassembly of reaction centres during recovery. In addition, ROS detoxification was activated via the expression of the peroxiredoxine enzyme (*ahpC*) and the glutathione synthesis genes. The generation of glutathione starts at T0H via the formation of glutamate from proline by *hyuA*, from aspartate by aspartate aminotransferase *(aat1)*, from 1-pyrroline-5-carboxylate by (*putA*), and from 2-oxoglutarate via GLDH (see Fig 8B). Glutamate synthesis via the GS/GOGAT cycle was repressed. The final synthesis of glutathione from glutamate occurred via glutathione (GSH) synthase *(gshB)*, which continued during recovery (see Fig 8B). Chaperones (*dnaK1*, *dnaK2*, *hspA*, *cbpA*) and proteases (*clpB2*) were also significantly induced during this stage, to remove damaged proteins. The free amino-acids released from protein degradation, likely lead to the production of urea, and the urease (*ureABC)* activity, transforming urea to ammonium, was induced. In parallel *Arthrospira* enhance some genes related to DNA repair system (*uvrBCD* for nucleotide excision and repair, *ruvB* resolving holiday junction, and *recJ*, *dnaG and mod* genes). The DNA-repair mechanism of *Arthrospira* included also enzymatic restriction modification (*hsdr*) and endonucleases. (B) During the **later phase** *Arthrospira* cells try to **recover from the damage; which lead to a slowly restored expression** of the genes related to photosynthesis and energy production, carbon fixation via the CBBn cycle and gluconeogenesis, TCA cycle. Expression of the hydrogenase genes (*hypA1*, *hypB1and hoxW*). Metal chaperone proteins HypA and HypB are required for the nickel insertion step of [NiFe]-hydrogenase maturation. In parallel slight reactivation of amino-acid transport (*aapJPQ*, *argGHJ*, *iaaA*) occurred. The genes for import of taurine (*tauABC*) known as organic sulphur and amino source were highly induced. The restoration of agmatinase, the key enzyme of agmatine hydrolysis was seen in recovery period. ROS detoxification was maintained efficiently via the expression for glutathione biosynthesis (GSH). Few genes related to protein damage clean up (proteases and chaperones) and DNA repair maintained their expression during recovery. The expression of gene cluster *arhABCDEF*, enriched during recovery, was seen.

The thiol group is very reactive, and quickly neutralizes radicals, such hydrogen peroxide, singlet oxygen and hydroxyl radicals. Several studies report a significant role of the such small antioxidants molecules in protecting proteins against oxidation after irradiation and as such preserving the enzymatic functions needed for DNA repair and cell recovery [64–66] (Fig 8).

In line with the detoxification systems described above, gene expression results demonstrate an increase in trans-membrane transport of redox-active metals. It is well known that metals such as iron (Fe), manganese (Mn), magnesium (Mg), and copper (Cu) are essential cofactors for the operation of the oxygenic photosynthetic electron transfer apparatus [67]. Higher levels of these metals are found in cyanobacteria compared to non-photosynthetic bacteria [68].

For instance an induced expression of genes coding for the FeoA and FeoB proteins was observed. They are part of the Feo system, mediating the ferrous iron Fe(II) import. Fe(II) is an important cofactor of redox active enzymes with iron-sulfur (Fe-S) cluster and cytochromes, which can help the cell in ROS detoxification. Furthermore, we observed the induced expression of *corA* and *mgtC* genes involved in magnesium uptake. Mg^2+^ is a critical component of the chlorophyll rings. The increased expression of Mg^2+^ uptake is likely in support of photosynthesis recovery.

In addition, the observed induced expression of *cutA* gene is likely related to copper uptake and general copper metabolism, potentially linked to Cyt C biogenesis [69]. Copper is an essential trace element and serves as an electron donor/acceptor by alternating between the redox states Cu(I) and Cu(II) in many copper-containing proteins, such as cytochrome c oxidase, the terminal electron acceptor of the respiratory chain, plastocyanin and superoxide dismutase, required for defence against oxidative damage.

Nevertheless, despite the fact that metals play a key role in oxygenic photosynthesis process, they can pose at the same time a major risk via the generation of free radicals (ROS). Therefore, metal transport and storage are tightly regulated to ensure adequate supply and to protect against oxidative damage, a process called metal homeostasis. The proliferation of all photosynthetic organisms depends on this delicate balance between the metal requirements and oxidative damage [70].

The activation of such ROS detoxification response normally involves different sensors, signal transduction systems and transcriptional regulators. Molecules with PAS and GAF domains serve as specific sensors that react to oxidative stress, light, oxygen and many other signals [71, 72], and were also overexpressed at RNA level in *Arthrospira* sp. PCC 8005 in response to ionising radiation. Gene expression showed also the induction of the *cry*-DASH gene well known as photo-sensor for UV-A light induced photo-tactic movement [29]. A recent study reported that Syn-*cry*, the *cry*-DASH gene from the cyanobacterium *Synechosystis sp*. PCC 6803, is required for efficient restoration of photosystem activity following UV-B and PAR induced photo-damage [73]. Also, the transcription of the genes for synthesis of the secondary messenger cyclic diguanylate (c-di-GMP) was induced. The aconitase enzyme (in TCA cycle), which showed an increased transcription upon irradiation (3200 Gy), has been reported to play a role in transcriptional regulation of ROS detoxification processes [74]. It has been proposed that aconitase, uses its iron sulphur cluster [4Fe-4S] as ROS sensor [75]. The FeS assembly machinery SUF (sulphur assimilation) capable of synthesising [Fe-S] clusters displayed an induced expression upon irradiation (Fig 8).

Despite antioxidant systems, radiation usually still damages lipids, proteins and DNA, which needs to be cleaned up or repaired within the cell to allow survival and proliferation. In cyanobacteria, lipids present in the thylakoids contain a high percentage of polyunsaturated fatty acid (PUFA) residues and are thus susceptible to peroxidation [76]. Gene expression showed induction of esterase/lipase activity that might be involved in degradation of damage lipids. In the meantime, desaturase genes (*des*) were significantly repressed. Proteolysis and the import of exogenous peptides and amino-acids are among the metabolic properties reported for *Deinococcus* to overcome oxidative stress [39]. Also *Arthrospira* seemed to increased protease activity during early response which was maintained in the recovery period. Together with proteases, also heat shock protein and chaperone genes were overexpressed, well known to be involved in stress response [77].

In bacteria such as *E*. *coli* and *B*. *subtilis* repair of radiation-induced DNA-damage is activated via the SOS system. Two key proteins regulate the SOS system: RecA and transcriptional repressor LexA, where the former activates auto-cleavage of the latter to induce SOS response. However, in the study of Narumi et al. [78] the authors demonstrate the non-involvement of LexA in the *recA* induction in *D*. *radiodurans* following gamma radiation. The *recA* gene and several other DNA repair genes in *Deinococcus* species are not regulated by LexA but by another transcriptional repressor protein, that is inactivated by a specific metalloprotease following gamma radiation [79]. In *Synechocystis* sp. PCC6803, LexA might have adopted a new function and no longer be in charge of the SOS response genes. From microarray gene profiling analysis it was concluded that in this species LexA might be involved in carbon metabolism [80, 81]. *Arthrospira* sp. PCC 8005 genome lacks the gene of the LexA repressor. In addition, the cells did not induce the *recA* gene in this study. It might be possible that a large amount of RecA protein is constantly present in the cell, even in the absence of DNA damage, and therefore is not induced by radiation. A similar observation was reported in *Helicobacter pylori* [82], showing no induced expression of the RecA protein following exposure to UV (10 J/m^2^) or gamma radiation (75 Gy). The only DNA-repair pathway clearly activated in *Arthrospira* sp. PCC 8005 in a dose dependent manner at early response was the nucleotide excision and repair (NER) mechanism (*uvrBCD* genes), responsible for the repair of single strand breaks [83]. In addition, *Arthrospira* sp. PCC 8005 activated several genes involved in DNA Restriction and Modification. These findings would suggest that the main effort is going to single strand break repair. But it does not exclude that there is double strand damage and repair or that the true mechanism of double strand DNA repair systems of this organism are yet to be characterised.

The most pertinent finding is the high expression of the gene cluster *arhABCDEF*, the only set of genes that was highly induced from start and throughout the full recovery. As reported before, the cluster was overexpressed both on RNA and protein level in response to irradiation [12]. The gene set was induced in dose dependent manner after exposure of *Arthrospira* sp. PCC 8005 to lethal doses (e.g: 3200 Gy and 5000 Gy) of ^60^Co gamma rays [12]. So far these proteins were not differentially induced in *Arthrospira* sp. PCC 8005 in response to other stresses, such as nitrogen deprivation [84] or light/dark growth transition [85]. These findings suggest a clear correlation between this gene set and the resistance of *Arthospira* sp. PCC 8005 to gamma rays. Although, no clear function could be assigned to these genes yet, it cannot be excluded that they are involved in DNA repair. Two of the five genes (i.e. *arhC* and *arhB*) show a significant homology with the conserved domain of chromosome segregation proteins (SMC). In eukaryotic cells SMC proteins are responsible for chromosome condensation, segregation, cohesion and DNA recombination repair [86]. SMC-like proteins are also present in Bacteria and Archea and perform essential functions in a variety of chromosome dynamics, such as chromosome compaction, segregation, and DNA repair [87]. SMC-like genes that exist in bacteria include *recN* and *sbcC*, which catalyse protein assembly at replication forks. The genes *recN* and *sbcC* are also present in PCC 8005, but they were not differentially induced in response to ionising radiation. This finding urge for additional studies, to further dissect the clear function of these *arhABCDEF* genes and proteins.

## Conclusion

The response of the cyanobacteria *Arthrospira* to an acute exposure to gamma rays involves a fast switch from an active growth state to a kind of 'survival state' during which the cells put photosynthesis and carbon and nitrogen assimilation on hold and activate pathways for cellular protection, detoxification, and repair. The cellular ROS detoxification and redox regulation of *Arthrospira* is mainly based on glutathione and metal homeostasis to protect essential lipids, proteins and DNA from oxidation. Beyond protein protection from oxidation, *Arthrospira* cells activate chaperones and proteases for de-folding of aggregated proteins and removal of the dysfunctional ones. DNA damage repair does not rely on the classical SOS response system. However, the radiation resistance of *Arthrospira* likely involves also some genes unique for *Arthrospira* with unknown functions but which are highly and specifically induced in response to radiation, in a dose dependent manner. The higher the radiation dose, the more pronounced this shock response and acclimation to survival state is induced. This probably makes it for the cells more difficult to revert from survival to active growth state, resulting in a longer delay in photosynthetic recovery and slower growth rates after exposure to higher radiation doses.

## Supporting Information

S1 Fig Growth curves of *Arthrospira* sp. PCC 8005 following exposure to different doses of gamma rays. Data represent mean of three independent biological replicates (n = 3), and error bars present the standard error of the mean (SEM). Two asterisks indicates that the growth rate value for the irradiated sample was significant (p<0.01) different from the value of the corresponding non-irradiated control. Three asterisk indicate a value which is highly significant (p<0.001).
(TIF)

S1 TableSpecific growth rate for the *Arthrospira* sp. PCC 8005 cultures grown after exposure to different doses of gamma rays.For each time interval between 2 time points, the specific growth rate (expressed as in increase in optical density measured at 750nm) was calculated with following formula: $\mu =\frac{\text{ln}\left(\text{OD}750\;\text{at}\;\text{t}2\right)-\text{ln}\left(OD750\;at\;t1\right)}{t2-t1}$. The last row presents the maximum growth rate obtained for each radiation dose. Data represent mean of three independent cultures (n = 3). An asterisk indicates a value for the irradiated sample which is significant (p<0.05) different from the value of the corresponding non-irradiated control. Three asterisk indicate a value which is highly significant (p<0.001).(DOCX)

S2 Table Genes differentially expressed by *Arthrospira* sp. PCC 8005 during the early emergency response after irradiation. Genes belong to clusters 3, 5, 4 and 6. Clustering was done on genes having Log_2_FC was equal or higher than 1 for up-regulated genes, and equal or lower than-1 for the down regulated ones and a p-value corrected for multiple testing lower than 0.05, in either one of the 9 conditions.
(DOCX)

S3 Table Genes silenced in *Arthrospira* sp. PCC 8005 during the early emergency response after irradiation. Genes belong to clusters 1, 2, 7, 8 and 9. Clustering was done on genes having Log_2_FC was equal or higher than 1 for up-regulated genes, and equal or lower than-1 for the down regulated ones and a p-value corrected for multiple testing lower than 0.05, in either one of the 9 conditions.
(DOCX)

S4 Table Genes expressed in *Arthrospira* sp. PCC 8005 during recovery after irradiation, belonging to clusters 1, 2, 7, 8 and 9. As input the fold changes of those genes are having Log_2_FC was equal or higher than 1 for up-regulated genes, and equal or lower than-1 for the down regulated ones and a p-value corrected for multiple testing lower than 0.05 in either one of the 9 conditions.
(DOCX)

S5 Table Genes expressed in Arthrospira sp. PCC 8005 during emergency response and throughout recovery after irradiation, belonging to cluster 8 and 9. As input the fold changes of those genes are having Log2FC was equal or higher than 1 for up-regulated genes, and equal or lower than-1 for the down regulated ones and a p-value corrected for multiple testing lower than 0.05 in either one of the 9 conditions.
(DOCX)
