# Supplementary figures and images for: Temporal Gene Expression of the Cyanobacterium Arthrospira in Response to Gamma Rays
Source: PLoS One. 2015 Aug 26;10(8):e0135565. doi: 10.1371/journal.pone.0135565 (PMC4550399; doi:10.1371/journal.pone.0135565)

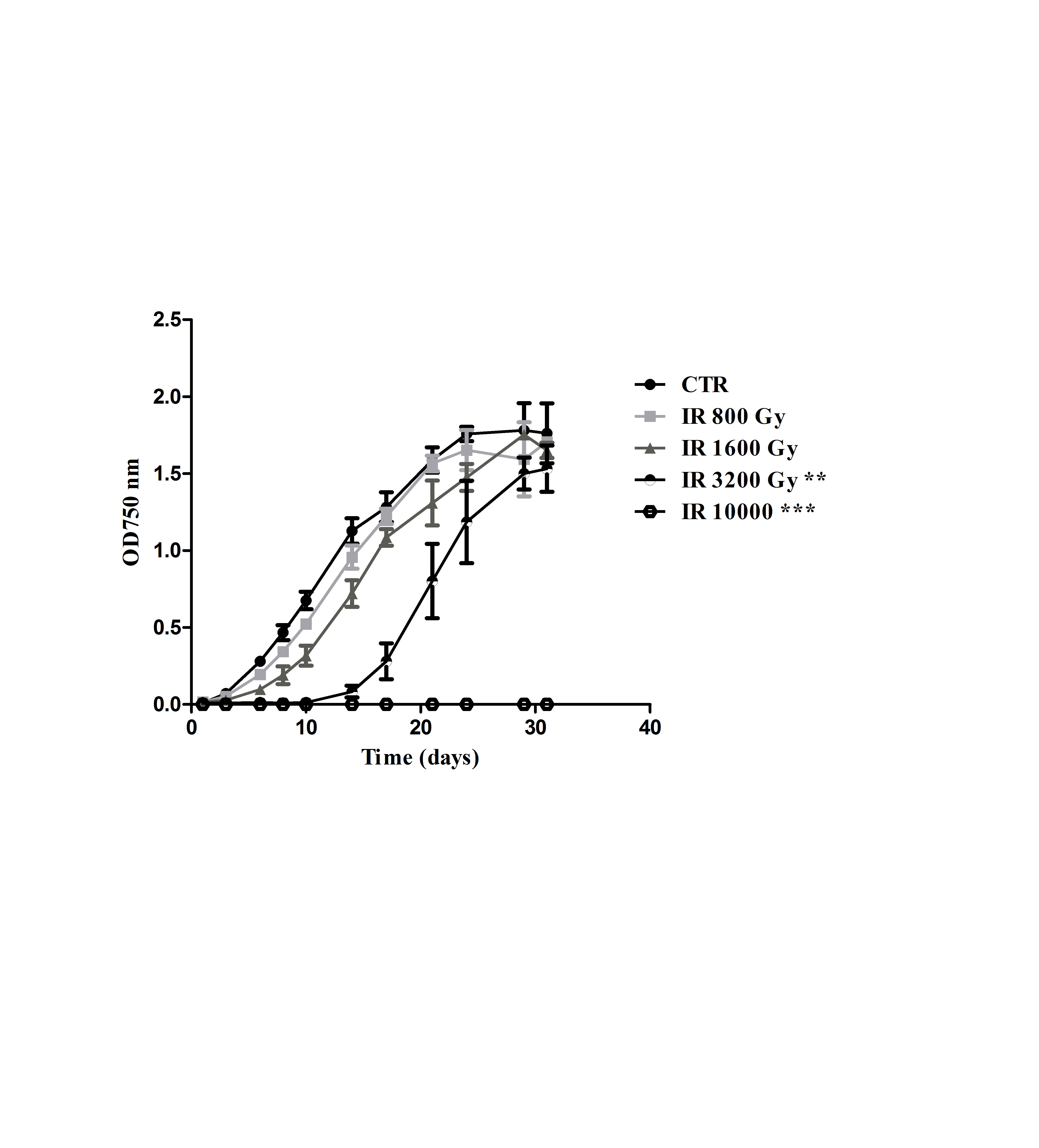

Supplement: S1 Fig — Data represent mean of three independent biological replicates (n = 3), and error bars present the standard error of the mean (SEM). Two asterisks indicates that the growth rate value for the irradiated sample was significant (p<0.01) different from the value of the corresponding non-irradiated control. Three asterisk indicate a value which is highly significant (p<0.001). (TIF) [file pone.0135565.s001.tif]
