# Supplementary material for: Temporal Gene Expression of the Cyanobacterium Arthrospira in Response to Gamma Rays
Source: PLoS One. 2015 Aug 26;10(8):e0135565. doi: 10.1371/journal.pone.0135565 (PMC4550399; doi:10.1371/journal.pone.0135565)
Supplement: S1 Table — For each time interval between 2 time points, the specific growth rate (expressed as in increase in optical density measured at 750nm) was calculated with following formula: μ=ln⁡(OD750att2)−ln(OD750att1)t2-t1. The last row presents the maximum growth rate obtained for each radiation dose. Data represent mean of three independent cultures (n = 3). An asterisk indicates a value for the irradiated sample which is significant (p<0.05) different from the value of the corresponding non-irradiated control. Three asterisk indicate a value which is highly significant (p<0.001). (DOCX) [file pone.0135565.s002.docx]

| Time intervals (Days) | CTR  (n=3) | 800 Gy  (n=3) | 1600 Gy  (n=3) | 3200 Gy  (n=3) |
| --- | --- | --- | --- | --- |
| 1-3 | **0,956** | **0,686** | 0,284 | -0,591 |
| 3-6 | 0,463 | 0,440 | **0,406** | 0,326 |
| 6-8 | 0,255 | 0,286 | 0,319 | -0,168 |
| 8-10 | 0,186 | 0,212 | 0,279 | 0,231 |
| 10-14 | 0,128 | 0,150 | 0,212 | 0,435 |
| 14-17 | 0,042 | 0,082 | 0,140 | **0,446** |
| 17-21 | 0,054 | 0,062 | 0,044 | 0,315 |
| 21-24 | 0,034 | 0,016 | 0,042 | 0,152 |
| 24-29 | 0,0008 | -0,011 | 0,035 | 0,058 |
| 29-31 | -0,007 | 0,046 | -0,030 | 0,007 |
| 31-33 | - | - | - | -0,009 |
| Lag time (µ = 0)(days) | 0 | 0 | 0 | 8** |
| Exponential phase (µ>0)(days) | 29 | 24 | 29 | 29 |
| Maximum growth rate µ_max_  (∆OD750*Day^-1^) | **0,956**  ±0,149 | **0,686**  ±0,235 | **0,406***  ±0,106 | **0,446***  ± 0,116 |
