# Supplementary material for: Temporal Gene Expression of the Cyanobacterium Arthrospira in Response to Gamma Rays
Source: PLoS One. 2015 Aug 26;10(8):e0135565. doi: 10.1371/journal.pone.0135565 (PMC4550399; doi:10.1371/journal.pone.0135565)
Supplement: S2 Table — Genes belong to clusters 3, 5, 4 and 6. Clustering was done on genes having Log2FC was equal or higher than 1 for up-regulated genes, and equal or lower than-1 for the down regulated ones and a p-value corrected for multiple testing lower than 0.05, in either one of the 9 conditions. (DOCX) [file pone.0135565.s003.docx]

| Emergency response | Gene name | Protein function | 800 T0H | 800 T2H | 800 T5H | 1600 T0H | 1600 T2H | 1600 T5H | 3200 T0H | 3200  T2H | 3200  T5H |
| --- | --- | --- | --- | --- | --- | --- | --- | --- | --- | --- | --- |
| Antioxidants | | | | | | | | | | | |
| ARTHROv5_30341 | *ahpC* | putative peroxiredoxin | 2,10 | 1,35 | 0,23 | 1,73 | 1,15 | 0,28 | 1,20 | 1,06 | 0,41 |
| Glutamate synthesis | | | | | | | | | | | |
| ARTHROv5_10456 | *hyuA* | 5-oxoprolinase | 3,79 | -1,34 | 0,94 | 3,89 | -0,10 | 0,00 | 3,89 | 1,80 | 0,45 |
| ARTHROv5_41057 | *proA1* | Gamma-glutamyl phosphate reductase | 2,32 | 1,28 | 1,44 | 2,69 | 1,44 | 1,32 | 2,87 | 2,05 | 1,35 |
| ARTHROv5_30794 | *putA* | proline dehydrogenase and 1-pyrroline-5 carboxylate dehydrogenase | 0,98 | 0,17 | 0,24 | 1,26 | 0,21 | 0,26 | 1,54 | 0,48 | 0,16 |
| ARTHROv5_10357 | *pep* | prolyl endopeptidase (PE) | 2,03 | 1,18 | 1,05 | 2,32 | 1,24 | 0,93 | 2,52 | 1,78 | 1,03 |
| ARTHROv5_30084 | *aat1* | Aspartate aminotransferase | 2,35 | 0,96 | 1,16 | 2,97 | 1,19 | 1,16 | 3,29 | 1,51 | 1,14 |
| Redox Metal homeostasis | | | | | | | | | | | |
| ARTHROv5_10584 | *copA1* | copper transport | -0,39 | -0,41 | -0,04 | 0,13 | 0,02 | 0,07 | 1,40 | 0,03 | -0,17 |
| ARTHROv5_40724 | *cutA* | copper transport | 0,64 | -0,04 | 0,50 | 1,06 | 0,14 | 0,38 | 1,67 | 0,50 | 0,29 |
| ARTHROv5_60812 | *corA* | magnesium/nickel/cobalt transport | 0,67 | 0,39 | 0,46 | 0,82 | 0,54 | 0,35 | 2,17 | 1,17 | 0,26 |
| ARTHROv5_41061 | *mtgC* | magnesium transport | -0,16 | -0,16 | -0,34 | 0,69 | -0,17 | -0,60 | 1,55 | 0,12 | -0,74 |
| ARTHROv5_11739 | *znuA* | zinc transport | 1,25 | 0,06 | 0,16 | 1,10 | 0,19 | 0,20 | 1,00 | 0,60 | -0,18 |
| ARTHROv5_61253 | *cobB* | cobyrinic acid | -0,28 | -0,14 | -0,45 | -0,23 | -0,07 | -0,36 | 1,11 | 0,01 | -0,66 |
| ARTHROv5_30441 | *cbiO1* | cobalt transport | 0,87 | -0,21 | 0,06 | 1,25 | -0,16 | 0,12 | 1,36 | 0,08 | -0,01 |
| ARTHROv5_10935 | *cbiQ2* | cobalt transport | 2,20 | 1,32 | 1,17 | 2,39 | 1,28 | 1,11 | 2,70 | 1,99 | 1,21 |
| ARTHROv5_61130 |  | potassium transport | 1,95 | 0,51 | 0,61 | 1,83 | 0,46 | 0,63 | 2,06 | 0,81 | 0,76 |
| ARTHROv5_40647 | *feoA* | ferrous iron transport | 1,76 | -0,25 | 0,01 | 0,81 | 0,21 | -0,3 | -0,95 | 0,65 | -1,00 |
| ARTHROv5_40648 | *feoB* | ferrous iron transport | 1,41 | -0,34 | -0,01 | 0,45 | 0,03 | -0,21 | -0,59 | 0,32 | -0,64 |
| ARTHROv5_60903 |  | ferric iron transport | 4,59 | 0,74 | 0,51 | 4,14 | 0,61 | 0,22 | 3,38 | 1,63 | 0,07 |
| ARTHROv5_60473 | *fur* | ferric iron uptake regulation | -0,39 | 0,06 | 0,47 | 0,94 | 0,75 | 0,49 | 1,98 | 1,32 | 0,08 |
| ARTHROv5_61180 | *isiA* | iron stress-induced chlorophyll-binding protein (CP43') | 4,52 | 2,15 | 1,04 | 3,81 | 2,28 | 0,79 | 3,12 | 2,58 | 0,88 |
| ARTHROv5_30590 |  | transcriptional regulator with CopG/Arc/MetJ | 3,27 | 2,29 | 2,07 | 4,16 | 2,19 | 2,15 | 4,04 | 2,77 | 2,64 |
| ARTHROv5_41184 | *ompA* | Outer membrane porin | 1,28 | -0,10 | -0,21 | 2,66 | 0,18 | 0,29 | 3,31 | 0,24 | -0,24 |
| ARTHROv5_41185 | *ompA* | Outer membrane porin | 3,25 | 0,55 | 0,17 | 4,24 | 0,79 | 0,82 | 4,22 | 1,02 | 0,51 |
| ARTHROv5_60877 |  | putative permease | 0,70 | 0,16 | 0,03 | 1,20 | 0,08 | 0,14 | 1,47 | 0,47 | 0,00 |
| ARTHROv5_11605 |  | putative permease | 1,99 | 0,90 | 1,26 | 2,44 | 1,01 | 1,18 | 2,84 | 1,60 | 1,36 |
| Protein damage clean up | | | | | | | | | | | |
| ARTHROv5_10934 |  | putative metallopeptidase | 0,92 | 0,73 | 0,36 | 1,22 | 0,54 | 0,57 | 2,06 | 0,86 | 0,3 |
| ARTHROv5_40599 | *patG* | Subtilisin-like protease | 2,30 | 1,12 | 1,22 | 2,19 | 1,19 | 1,20 | 1,93 | 1,04 | 1,04 |
| ARTHROv5_30014 | *dnaK1* | Chaperone protein | 1,84 | 0,76 | 1,01 | 2,56 | 0,95 | 0,88 | 3,2 | 1,59 | 1,02 |
| ARTHROv5_11999 | *dnaK2* | Hsp70, co-chaperone with DnaJ | 1,04 | -0,20 | -0,37 | 0,65 | -0,42 | -0,12 | 0,10 | -0,45 | -0,19 |
| ARTHROv5_61125 | *hspA* | heat shock protein A | 1,56 | 0,29 | 0,27 | 1,43 | 0,42 | 0,45 | 1,40 | 0,70 | 0,46 |
| ARTHROv5_61127 | *cbpA* | curved DNA-binding protein, DnaJ homologue | 1,71 | 0,25 | 0,25 | 1,58 | 0,33 | 0,31 | 1,68 | 0,59 | 0,49 |
| ARTHROv5_41259 | *clpB2* | protein disaggregation chaperone | 1,67 | -0,26 | -0,09 | 1,51 | -0,13 | 0,07 | 0,94 | -0,12 | 0,03 |
| ARTHROv5_11765 | *sufR* | iron-sulphur cluster biosynthesis transcriptional regulator SufR | 1,25 | 0,07 | 0,29 | 1,22 | 0,12 | 0,41 | 1,32 | 0,33 | 0,64 |
| ARTHROv5_11769 | *sufS* | cysteine desulfurase SufS | 1,30 | 0,04 | 0,38 | 1,18 | 0,17 | 0,21 | 1,33 | 0,78 | 0,33 |
|  |  |  |  |  |  |  |  |  |  |  |  |
| DNA damage repair | | | | | | | | | | | |
| ARTHROv5_11714 | *nudE* | putative ADP-ribose pyrophosphatase | 0,34 | 0,26 | 0,36 | 1,93 | 0,45 | 0,37 | 3,59 | 0,82 | 0,85 |
| ARTHROv5_40086 | *mutT* | NUDIX hydrolase | 1,27 | 0,73 | 0,73 | 1,92 | 0,77 | 0,62 | 2,45 | 1,26 | 0,77 |
| ARTHROv5_20108 | *recJ* | Single-strand-DNA-specificexonuclease | 1,46 | 0,26 | 0,40 | 2,08 | 0,57 | 0,33 | 1,66 | 0,97 | 0,18 |
| ARTHROv5_40732 | *uvrB* | exconulease of nucleotide excision repair | 2,02 | 1,01 | 1,32 | 2,60 | 1,19 | 1,24 | 2,73 | 1,84 | 1,17 |
| ARTHROv5_60258 | *uvrC* | UvrABC system protein C | 1,48 | 0,81 | 0,30 | 2,19 | 0,80 | 0,52 | 2,11 | 1,20 | 0,20 |
| ARTHROv5_41027 | *uvrD* | DNA helicase, UvrD/REP | 2,84 | 1,46 | 1,56 | 3,87 | 1,76 | 1,67 | 4,03 | 2,43 | 1,81 |
| ARTHROv5_60750 | *helD* | putative DNA helicase, UvrD-family | 1,13 | -0,17 | -0,17 | 1,15 | -0,22 | -0,10 | 0,92 | -0,13 | -0,48 |
| ARTHROv5_30188 |  | Helicase-like protein | 1,88 | 0,51 | 0,27 | 1,96 | 0,56 | 0,55 | 2,32 | 0,74 | 0,57 |
| ARTHROv5_11763 | *ruvB* | ATP-dependent DNA helicase | 1,12 | 0,08 | -0,22 | 0,98 | -0,04 | -0,04 | 1,57 | 0,57 | -0,18 |
| ARTHROv5_10675 | *dnaG* | DNA primase | 2,06 | 0,70 | 0,81 | 2,35 | 0,83 | 1,03 | 1,81 | 0,94 | 1,21 |
| DNA modification and protection | | | | | | | | | | | |
| ARTHROv5_30008 | *mod* | Modification methylase | 1,23 | 0,38 | 0,33 | 1,83 | 0,45 | 0,61 | 1,09 | 0,36 | 0,59 |
| ARTHROv5_30623 | *hsdR1* | Type I site-specific deoxyribonuclease, HsdR | 4,19 | 2,50 | 2,58 | 4,47 | 2,84 | 2,61 | 4,63 | 3,53 | 2,85 |
| ARTHROv5_30624 | *hsdR2* | Type I site-specific deoxyribonuclease, HsdR | 3,64 | 2,02 | 2,31 | 4,09 | 2,40 | 2,38 | 4,31 | 3,03 | 2,65 |
| ARTHROv5_30625 | *hsdR3* | Type I site-specific deoxyribonuclease, HsdR | 2,51 | 0,95 | 0,90 | 3,09 | 1,38 | 0,93 | 3,37 | 2,03 | 0,98 |
| ARTHROv5_60368 |  | Type II DNA modification enzyme | 1,85 | 0,54 | 0,65 | 2,20 | 0,62 | 0,50 | 1,78 | 1,02 | 0,78 |
| ARTHROv5_40255 | *asp8005ORF5800M* | Type II DNA modification methyltransferase | 1,18 | 0,31 | 0,31 | 1,93 | 0,45 | 0,50 | 1,80 | 0,82 | 0,25 |
| ARTHROv5_30352 | *asp8005ORF0359* | Type II restriction enzyme | 2,68 | 1,06 | 0,80 | 3,09 | 0,57 | 1,25 | 2,21 | 0,42 | 1,59 |
| ARTHROv5_50004 | *pvuIIR* | Type II restriction enzyme | 2,67 | 0,45 | 0,31 | 2,83 | 0,26 | 0,21 | 3,07 | 0,50 | 0,16 |
| DNA modification – FAX elements | | | | | | | | | | | |
| ARTHROv5_10168 | *faxB1* | unknown phage of the genus *Arthrospira*, protein B | 0,62 | -0,23 | -0,13 | 0,73 | 0,20 | 0,56 | 1,47 | 0,11 | -0,10 |
| ARTHROv5_30751 | *faxB1* | unknown phage of the genus *Arthrospira*, protein B | 0,82 | -0,05 | 0,27 | 0,93 | 0,40 | 0,74 | 2,06 | 0,46 | 0,29 |
| ARTHROv5_30553 | *faxK1f3* | unknown phage of the genus *Arthrospira*, protein K | 3,16 | 1,32 | 0,69 | 2,26 | 1,66 | 1,36 | 1,58 | 1,57 | 1,20 |
| ARTHROv5_40353 | *faxK4f2* | unknown phage of the genus *Arthrospira*, protein K | 3,28 | 1,74 | 1,25 | 2,25 | 1,83 | 1,60 | 1,87 | 1,86 | 1,72 |
| ARTHROv5_30731 | *faxO8f* | unknown phage of the genus *Arthrospira*, protein O | 2,31 | 0,79 | 0,73 | 2,43 | 1,21 | 1,05 | 2,55 | 1,39 | 1,04 |
| ARTHROv5_30730 | *faxP8* | unknown phage of the genus *Arthrospira*, protein P | 1,21 | 0,40 | 0,45 | 1,85 | 0,37 | 0,78 | 2,65 | 0,51 | 0,79 |
| T/TA systems | | | | | | | | | | | |
| ARTHROv5_11210 | *yefM* | putative antitoxin of toxin-antitoxin | 2,71 | 1,53 | 1,57 | 3,43 | 1,41 | 1,69 | 3,62 | 1,94 | 1,71 |
| ARTHROv5_11211 | *yoeB* | putative toxin of toxin-antitoxin | 4,88 | 2,50 | 3,00 | 5,41 | 2,52 | 3,04 | 5,53 | 2,93 | 3,28 |
| ARTHROv5_12008 | *mazF9* | mRNA interferase | 2,64 | 0,75 | 2,11 | 2,97 | 1,12 | 1,93 | 3,24 | 1,54 | 2,15 |
| ARTHROv5_12009 |  | Toxin/antitoxin | 2,54 | 0,79 | 1,54 | 2,75 | 1,26 | 1,35 | 3,23 | 1,96 | 1,58 |
| DNA modification – CRISPER elements | | | | | | | | | | | |
| ARTHROv5_40678 | *cas1* | CRISPR-associated endonuclease Cas1 | 1,87 | 0,54 | 0,67 | 2,59 | 0,77 | 0,80 | 2,95 | 1,08 | 0,74 |
| ARTHROv5_40676 | *cas2* | CRISPR-associated endoribonuclease Cas2 | 2,13 | 0,56 | 0,68 | 2,64 | 0,66 | 0,91 | 2,97 | 0,98 | 1,07 |
| ARTHROv5_40718 |  | CRISPR-associated RAMP protein, Csm3 | 2,48 | 1,09 | 1,14 | 2,47 | 1,31 | 1,30 | 1,55 | 1,48 | 1,09 |
| ARTHROv5_40717 |  | CRISPR-associated RAMP protein, Csm4 | 2,67 | 1,30 | 1,18 | 2,45 | 1,36 | 1,44 | 1,66 | 1,56 | 1,15 |
| C-Storage & protection molecules | | | | | | | | | | | |
| ARTHROv5_11781 | *sigE* | Transcriptional regulator degradation of glycogen | 1,71 | 0,24 | 0,66 | 1,83 | 0,44 | 0,74 | 1,70 | 0,29 | 0,45 |
| ARTHROv5_41060 | *treS* | putative Trehalose synthase | 1,39 | 0,32 | 0,43 | 1,54 | 0,23 | 0,29 | 1,58 | 0,67 | 0,30 |
| ARTHROv5_10500 | *phaC* | Poly(R)-hydroxyalkanoic acid biosynthesis | 2,00 | 0,60 | 1,00 | 2,14 | 0,79 | 1,02 | 2,36 | 0,95 | 1,06 |
| ARTHROv5_10936 | *cbiM2* | cobalamin biosynthesis protein | 1,52 | 0,87 | 0,68 | 1,69 | 0,88 | 0,54 | 1,96 | 1,36 | 0,57 |
| ARTHROv5_40927 | *cobW* | cobalamin biosynthesis protein | 2,45 | 1,68 | 1,86 | 2,98 | 1,87 | 1,82 | 3,16 | 2,30 | 1,82 |
| ARTHROv5_11097 |  | ABC-type sugar transport | 2,55 | 0,93 | 1,65 | 2,75 | 1,34 | 1,52 | 2,85 | 1,78 | 1,28 |
| ARTHROv5_60792 | *ugpB* | ABC-type sugar transport | 2,05 | 0,56 | 0,55 | 2,21 | 0,70 | 0,55 | 1,65 | 0,94 | 0,64 |
| Lipid Degradation | | | | | | | | | | | |
| ARTHROv5_50008 |  | Lipase class 3 | 1,22 | -1,73 | 1,84 | 2,84 | 1,14 | 1,76 | 2,14 | -0,49 | 1,54 |
| ARTHROv5_10744 |  | putative Esterase/lipase | 3,05 | 1,52 | 1,91 | 3,31 | 2,02 | 1,82 | 3,58 | 2,61 | 1,78 |
| Signal transduction | | | | | | | | | | | |
| ARTHROv5_10459 |  | putative diguanylate cyclase | 1,65 | -0,05 | 0,02 | 1,08 | 0,24 | -0,28 | 1,20 | 1,39 | -0,44 |
| ARTHROv5_10653 |  | putative diguanylate cyclase/phosphodiesterase | 0,96 | 0,34 | 0,14 | 1,20 | 0,07 | 0,28 | 1,53 | 0,53 | 0,62 |
| ARTHROv5_10654 |  | putative diguanylate cyclase/phosphodiesterase | 1,04 | 0,59 | 0,10 | 1,55 | 0,36 | 0,38 | 1,86 | 0,86 | 0,58 |
| ARTHROv5_10656 |  | putative diguanylate cyclase/phosphodiesterase | 0,86 | 0,41 | 0,27 | 1,95 | 0,30 | 0,56 | 2,70 | 0,83 | 0,80 |
| ARTHROv5_10963 | *cry* | Cryptochrome DASH | 2,16 | 0,84 | 1,30 | 2,11 | 0,96 | 1,36 | 2,15 | 1,60 | 1,28 |
| ARTHROv5_20097 |  | Putative diguanylate cyclase/ (GAF) sensor | 1,71 | -0,11 | 0,51 | 3,13 | 0,83 | 0,73 | 5,42 | 1,83 | 0,30 |
| ARTHROv5_20098 |  | Putative diguanylate cyclase (GGDEF domain) | 0,48 | -0,33 | 0,23 | 1,46 | 0,28 | 0,30 | 4,82 | 1,34 | 0,13 |
| ARTHROv5_30066 |  | putative diguanylate cyclase PleD-like (fragment) GGDEF domain | 1,19 | 0,60 | 0,33 | 2,44 | 0,61 | 0,54 | 2,87 | 0,92 | 0,60 |
| ARTHROv5_30396 |  | Putative diguanylate cyclase (GGDEF domain) | 1,92 | 0,71 | 0,76 | 1,85 | 0,55 | 0,93 | 2,14 | 1,06 | 0,80 |
| ARTHROv5_30397 |  | putative diguanylate cyclase PleD-like (fragment)GGDEF domain | 1,65 | 0,34 | 0,31 | 1,88 | 0,40 | 0,63 | 1,91 | 0,75 | 0,29 |
| ARTHROv5_40303 |  | putative Diguanylate kinase | 0,57 | 0,11 | 0,08 | 0,99 | 0,33 | 0,20 | 1,82 | 0,56 | 0,21 |
| ARTHROv5_50007 |  | putative diguanylate cyclase | 0,89 | -0,04 | 0,42 | 1,36 | 0,46 | 0,43 | 2,09 | 0,92 | 0,14 |
| ARTHROv5_50285 |  | putative Diguanylate cyclase/phosphodiesterase | 3,75 | 2,31 | 2,20 | 4,50 | 3,40 | 2,40 | 4,58 | 3,59 | 2,00 |
| Nitrogen metabolism |  |  |  |  |  |  |  |  |  |  |  |
| ARTHROv5_61139 | *lysE* | LysE/RhtB family amino acid efflux pump | 3,84 | 1,58 | 1,78 | 3,62 | 1,75 | 1,80 | 3,57 | 2,28 | 2,09 |
| ARTHROv5_30069 | *ureA* | Urease subunit gamma | 1,04 | 0,27 | 0,30 | 1,51 | 0,26 | 0,25 | 1,57 | 0,30 | 0,23 |
| ARTHROv5_30068 | *ureB* | Urease subunit beta | 0,94 | 0,28 | 0,28 | 1,48 | 0,17 | 0,22 | 1,42 | 0,32 | 0,29 |
| ARTHROv5_30067 | *ureC* | Urease subunit alpha | 1,11 | 0,28 | 0,30 | 1,68 | 0,30 | 0,25 | 1,78 | 0,46 | 0,33 |
| ARTHROv5_30070 | *ureD* | Urease accessory protein ureD | 1,84 | 0,64 | 0,80 | 2,64 | 0,70 | 0,82 | 3,02 | 1,02 | 1,14 |
| ARTHROv5_60622 | *nifU* | Nitrogen-fixation | 2,18 | 0,64 | 1,47 | 2,87 | 1,23 | 1,54 | 3,60 | 1,79 | 1,61 |
| ARTHROv5_40220 | *devA* | putative Heterocyst specific transporter | 2,53 | 0,97 | 1,50 | 3,24 | 1,16 | 1,45 | 3,31 | 1,84 | 1,47 |
| ARTHROv5_40318 | *rbsK* | Ribokinase (PPP) | 2,91 | 1,49 | 1,75 | 3,22 | 1,71 | 1,82 | 3,13 | 1,95 | 1,76 |
| ARTHROv5_30379 | *potB* | Polyamine transport | 1,06 | 0,60 | 0,42 | 1,54 | 0,92 | 0,58 | 1,98 | 1,14 | 0,61 |
| ARTHROv5_30377 | *potC* | Polyamine transport | 0,44 | -0,02 | -0,24 | 1,32 | 0,07 | 0,00 | 1,76 | 0,66 | -0,16 |
