# Supplementary material for: Temporal Gene Expression of the Cyanobacterium Arthrospira in Response to Gamma Rays
Source: PLoS One. 2015 Aug 26;10(8):e0135565. doi: 10.1371/journal.pone.0135565 (PMC4550399; doi:10.1371/journal.pone.0135565)
Supplement: S3 Table — Genes belong to clusters 1, 2, 7, 8 and 9. Clustering was done on genes having Log2FC was equal or higher than 1 for up-regulated genes, and equal or lower than-1 for the down regulated ones and a p-value corrected for multiple testing lower than 0.05, in either one of the 9 conditions. (DOCX) [file pone.0135565.s004.docx]

| Recovery period | Gene name | Gene function | 800 T0H | 800 T2H | 800 T5H | 1600 T0H | 1600 T2H | 1600 T5H | 3200 T0H | 3200  T2H | 3200  T5H |
| --- | --- | --- | --- | --- | --- | --- | --- | --- | --- | --- | --- |
| Phycobilisomes |  |  |  |  |  |  |  |  |  |  |  |
| ARTHROv5_10635 | *apcC* | Phycobilisome linker | -2,46 | -0,46 | -1,21 | -3,19 | -0,74 | -0,82 | -3,78 | -1,87 | -1,03 |
| ARTHROv5_10637 | *apcA* | Allophycocyanin alpha subunit | -1,38 | -0,24 | -0,62 | -1,64 | -0,40 | -0,28 | -2,43 | -1,21 | -0,36 |
| ARTHROv5_10636 | *apcB* | Allophycocyanin beta subunit | -2,13 | -0,50 | -1,15 | -2,49 | -0,77 | -0,69 | -3,41 | -1,98 | -0,90 |
| ARTHROv5_12132 | *apcF* | allophycocyanin beta subunit | -1,29 | -0,61 | -1,53 | -2,07 | -1,07 | -1,28 | -3,17 | -1,82 | -1,62 |
| ARTHROv5_11555 | *cpcC1* | Phycobilisome linker polypeptide, | -0,73 | -0,16 | -0,11 | -0,97 | -0,12 | -0,24 | -1,80 | -0,20 | -0,39 |
| ARTHROv5_11558 | *cpcE* | Phycocyanin alpha subunit | -0,67 | 0,05 | -0,12 | -0,90 | -0,00 | -0,08 | -1,62 | -0,20 | -0,04 |
| ARTHROv5_60720 | *cpcT* | Chromophore lyase | -1,16 | -0,21 | -0,86 | -1,89 | -0,50 | -0,70 | -1,78 | -0,79 | -0,98 |
| Haem |  |  |  |  |  |  |  |  |  |  |  |
| ARTHROv5_50123 | *hemC* | Porphobilinogen deaminase | -0,80 | -0,22 | -0,60 | -1,62 | -0,42 | -0,34 | -2,46 | -1,05 | -0,49 |
| ARTHROv5_60626 | *hemE* | Uroporphyrinogen decarboxylase | -1,08 | -0,18 | -0,77 | -1,95 | -0,49 | -0,53 | -2,64 | -1,11 | -0,67 |
| ARTHROv5_40397 | *cyoE* | Protoheme IX farnesyltransferase (heme O synthase) | -1,22 | 0,54 | -0,26 | -1,92 | 0,29 | 0,04 | -1,95 | -0,46 | 0,04 |
| Porphirin |  |  |  |  |  |  |  |  |  |  |  |
| ARTHROv5_11943 | *cobA* | Uroporphyrinogen-III C-methyltransferase | -0,99 | -0,35 | -0,16 | -1,34 | -1,15 | -0,15 | -1,71 | -1,36 | -0,41 |
| ARTHROv5_10139 | *hemG* | Protoporphyrinogen oxidase | -1,33 | -0,29 | -0,55 | -1,93 | -0,22 | -0,40 | -0,97 | -0,60 | -0,94 |
| ARTHROv5_60173 | *hemN1* | Oxygen-independent coproporphyrinogen | -2,77 | -0,37 | -0,97 | -2,64 | -0,80 | -0,57 | -1,89 | -1,31 | -0,85 |
| chlorophyll |  |  |  |  |  |  |  |  |  |  |  |
| ARTHROv5_30766 | *chlG* | Chlorophyll a synthase ChlG | -1,55 | 0,07 | -0,33 | -2,15 | 0,14 | -0,03 | -2,54 | -0,60 | -0,22 |
| ARTHROv5_11499 | *chlH* | Magnesium chelatase H subunit | -1,52 | 0,12 | -0,41 | -2,25 | -0,03 | -0,26 | -2,90 | -0,58 | -0,38 |
| ARTHROv5_40023 | *chlP* | geranylgeranyl reductase | -0,79 | -0,07 | -0,56 | -1,40 | -0,23 | -0,45 | -1,76 | -0,56 | -0,45 |
| ARTHROv5_40768 | *bchD* | Mg-protoporphyrin IX chelatase | -1,13 | -0,37 | -1,08 | -2,03 | -0,69 | -0,69 | -3,02 | -1,81 | -0,92 |
| Plastoquinone |  |  |  |  |  |  |  |  |  |  |  |
| ARTHROv5_10689 | *ndhA* | NADH:ubiquinone oxidoreductase, membrane subunit H | -0,90 | -0,17 | -0,31 | -1,56 | -0,30 | -0,19 | -2,11 | -0,75 | -0,29 |
| ARTHROv5_10690 | *ndhI* | NAD(P)H-quinone oxidoreductase subunit I | -1,49 | -0,25 | -0,62 | -2,14 | -0,38 | -0,40 | -2,70 | -1,04 | -0,61 |
| ARTHROv5_10691 | *ndhG* | NAD(P)H-quinone oxidoreductase chain 6 | -1,02 | -0,15 | -0,43 | -1,32 | -0,27 | -0,26 | -1,61 | -0,77 | -0,29 |
| ARTHROv5_10693 | *ndhE* | NADH:ubiquinone oxidoreductase, membrane subunit K | -1,38 | 0,08 | -0,51 | -2,03 | -0,29 | -0,34 | -2,30 | -0,79 | -0,45 |
| ARTHROv5_40057 | *ndhH* | NAD(P)H-quinone oxidoreductase chain H | -0,94 | -0,02 | -0,40 | -1,86 | -0,05 | -0,16 | -2,70 | -0,40 | -0,35 |
| ARTHROv5_40541 | *ndhF1* | NAD(P)H-quinone oxidoreductase chain 5 | -1,08 | 0,05 | -0,15 | -1,61 | -0,02 | 0,08 | -2,09 | -0,34 | 0,03 |
| ARTHROv5_40542 | *ndhD1* | NAD(P)H-quinone oxidoreductase chain 4 | -1,36 | 0,03 | -0,19 | -1,98 | -0,03 | 0,05 | -2,23 | -0,40 | 0,01 |
| ARTHROv5_60547 | *ndhJ* | NAD(P)H-quinone oxidoreductase subunit J | -0,83 | 0,01 | -0,25 | -1,30 | 0,13 | -0,16 | -1,76 | -0,19 | -0,36 |
| ARTHROv5_60549 | *ndhC* | NAD(P)H-quinone oxidoreductase subunit 3 | -0,62 | 0,14 | -0,20 | -1,19 | 0,04 | -0,12 | -1,92 | -0,18 | -0,32 |
| ARTHROv5_60715 | *ndhD4* | NAD(P)H-quinone oxidoreductase chain 4 | -1,49 | -0,57 | -0,71 | -2,40 | -0,68 | -0,54 | -2,29 | -1,46 | -0,87 |
| ARTHROv5_60716 | *ndhF* | NAD(P)H-quinone oxidoreductase subunit 5 | -0,92 | -0,47 | -0,53 | -2,02 | -0,43 | -0,36 | -2,69 | -1,22 | -0,71 |
| Cytochrome |  |  |  |  |  |  |  |  |  |  |  |
| ARTHROv5_61102 | *cydA* | Cytochrome bd ubiquinol oxidase, subunit I | -1,42 | 0,25 | -0,23 | -1,84 | 0,39 | -0,06 | -2,33 | 0,34 | -0,23 |
| ARTHROv5_61103 | *cydB* | Cytochrome bd ubiquinol oxidase, subunit II | -1,37 | 0,32 | -0,21 | -1,90 | 0,40 | -0,11 | -2,24 | 0,34 | -0,22 |
| ARTHROv5_40400 | *coxA* | Cytochrome c oxidase subunit I | -0,74 | 0,24 | -0,06 | -1,32 | 0,17 | 0,07 | -1,77 | -0,10 | -0,04 |
| ARTHROv5_40399 | *coxB* | Cytochrome c oxidase subunit II | -0,77 | 0,08 | -0,07 | -1,14 | 0,10 | 0,08 | -1,60 | -0,15 | -0,03 |
| ARTHROv5_40401 | *coxC* | Cytochrome c oxidase subunit III | -1,35 | 0,21 | -0,18 | -2,10 | 0,01 | -0,05 | -2,58 | -0,27 | -0,21 |
| ARTHROv5_40398 |  | putative Cytochrome oxidase assembly protein | -0,75 | 0,22 | -0,26 | -1,99 | 0,23 | -0,02 | -2,44 | -0,42 | -0,19 |
| ATP |  |  |  |  |  |  |  |  |  |  |  |
| ARTHROv5_60533 | *atpG2* | ATP synthase B' chain (Subunit II) | -1,30 | -0,74 | -1,23 | -2,04 | -1,16 | -1,11 | -2,54 | -1,83 | -1,45 |
| ARTHROv5_60534 | *atpF* | ATP synthase B chain (Subunit I) | -1,29 | -0,56 | -1,22 | -1,76 | -0,89 | -0,86 | -2,31 | -1,68 | -1,10 |
| ARTHROv5_60535 | *atpH* | ATP synthase delta chain; ATP synthase F1, delta subunit | -1,27 | -0,45 | -1,10 | -1,75 | -0,87 | -0,88 | -2,32 | -1,65 | -1,05 |
| CO_2_ fixation & CBB cycle |  |  |  |  |  |  |  |  |  |  |  |
| ARTHROv5_50352 | *cbbR* | RuBisCO operon transcriptional regulator | -0,94 | -0,16 | -0,76 | -1,71 | -0,61 | -0,47 | -2,32 | -1,03 | -0,60 |
| ARTHROv5_60714 |  | CO2 hydration protein | -1,98 | -0,61 | -0,89 | -2,58 | -0,82 | -0,68 | -3,14 | -1,70 | -0,93 |
| ARTHROv5_10443 | *pgi* | Glucose-6-phosphate isomerase | -0,78 | 0,17 | -0,48 | -1,47 | 0,06 | -0,42 | -1,32 | -0,18 | -0,70 |
| ARTHROv5_20037 | *pgk* | phosphoglycerate kinase | -0,92 | -0,36 | -0,53 | -1,46 | -0,45 | -0,48 | -2,49 | -0,73 | -0,71 |
| ARTHROv5_41419 | *xfp* | D-xylulose 5-phosphate/D-fructose 6-phosphate phosphoketolase | -0,98 | -0,25 | -0,44 | -1,84 | -0,60 | -0,40 | -2,79 | -0,71 | -0,37 |
| Carbon metabolism |  |  |  |  |  |  |  |  |  |  |  |
| ARTHROv5_30613 | *gap2* | Glyceraldehyde-3-phosphate dehydrogenase 2 | -0,75 | -0,44 | -0,71 | -1,34 | -0,77 | -0,60 | -2,61 | -1,35 | -0,80 |
| ARTHROv5_30667 | *gpmB* | phosphoglycerate mutase | -1,10 | 0,09 | -0,93 | -2,28 | -0,26 | -0,65 | -3,31 | -0,96 | -0,87 |
| ARTHROv5_30574 | *gpmI* | 2,3-bisphosphoglycerate-independent phosphoglycerate mutase | -1,08 | -0,12 | -0,69 | -2,00 | -0,28 | -0,34 | -2,41 | -0,93 | -0,58 |
| ARTHROv5_30318 | *pgm* | phosphoglucomutase | -0,91 | -0,35 | -0,84 | -1,58 | -0,63 | -0,75 | -2,21 | -1,18 | -0,90 |
| ARTHROv5_50271 | *glnB* | protein P-II | -1,80 | -0,28 | -0,70 | -2,31 | -0,85 | -0,43 | -2,73 | -1,49 | -0,56 |
| TCA cycle |  |  |  |  |  |  |  |  |  |  |  |
| ARTHROv5_12017 | *sdhA* | succinate dehydrogenase flavoprotein subunit | -1,16 | -0,53 | -0,59 | -1,83 | -0,26 | -0,42 | -2,18 | -0,85 | -0,73 |
| ARTHROv5_10965 | *sucC* | Succinyl-CoA ligase | -0,84 | -0,24 | -0,37 | -1,56 | -0,32 | -0,21 | -2,26 | -0,67 | -0,36 |
| ARTHROv5_10964 | *sucD* | succinyl-CoA synthetase | -1,13 | -0,10 | -0,27 | -1,55 | -0,37 | -0,15 | -2,03 | -0,66 | -0,24 |
| Fatty acid biosynthesis |  |  |  |  |  |  |  |  |  |  |  |
| ARTHROv5_11990 | *fabF2* | 3-oxoacyl-[acyl-carrier-protein] synthase 2 | -0,87 | 0,02 | -0,05 | -1,26 | 0,07 | 0,09 | -1,20 | -0,16 | 0,05 |
| ARTHROv5_30260 | *fabG1* | 3-oxoacyl-[acyl-carrier-protein] reductase | -1,89 | -0,37 | -1,03 | -2,26 | -0,72 | -0,81 | -2,98 | -1,27 | -1,02 |
| ARTHROv5_60058 | *fabG2* | 3-oxoacyl-[acyl-carrier-protein] reductase | -0,76 | -0,21 | -0,55 | -0,91 | -0,40 | -0,37 | -2,07 | -0,82 | -0,44 |
| ARTHROv5_30177 | *fabH* | 3-oxoacyl-[acyl-carrier-protein] synthase 3 | -1,46 | 0,11 | -0,85 | -2,22 | -0,37 | -0,75 | -1,80 | -1,10 | -0,83 |
| ARTHROv5_41232 | *fabZ* | (3R)-hydroxymyristoyl-[acyl-carrier-protein] dehydratase | -1,81 | -0,46 | -1,01 | -2,64 | -0,82 | -0,69 | -2,67 | -1,47 | -1,02 |
| ARTHROv5_60707 | *desA* | Delta(12)-fatty acid desaturase | -1,17 | 0,01 | -0,58 | -1,34 | -0,39 | -0,33 | -1,94 | -0,95 | -0,24 |
| ARTHROv5_40656 | *desD* | delta-6 fatty acid desaturase | -0,93 | 0,21 | -0,23 | -1,49 | 0,21 | 0,08 | -1,97 | -0,44 | -0,20 |
| Nitrogen metabolism |  |  |  |  |  |  |  |  |  |  |  |
| ARTHROv5_30825 | *ntcA* | Global nitrogen regulator | -0,53 | 0,12 | -0,20 | -1,08 | -0,01 | 0,00 | -1,35 | -0,34 | -0,01 |
| ARTHROv5_11376 | *amt1* | Ammonium/methylammonium permease | -1,93 | -1,73 | -1,45 | -2,21 | -2,54 | -1,18 | -2,86 | -3,33 | -1,53 |
| ARTHROv5_12133 | *glnA* | glutamine synthetase | -2,33 | -0,80 | -1,39 | -2,86 | -1,54 | -0,89 | -3,94 | -2,79 | -1,29 |
| ARTHROv5_50078 | *glsF* | Ferredoxin-dependent glutamate synthase, large subunit | -0,84 | -0,25 | -0,34 | -1,50 | -0,22 | -0,19 | -1,91 | -0,61 | -0,37 |
| ARTHROv5_60175 | *nthA1* | Nitrile hydratase alpha subunit | -2,62 | -0,58 | -0,83 | -3,11 | -0,99 | -0,32 | -3,65 | -1,97 | -0,55 |
| ARTHROv5_60176 | *nthB2* | Nitrile hydratase beta subunit | -2,53 | -0,51 | -0,89 | -3,14 | -1,19 | -0,42 | -3,65 | -2,13 | -0,65 |
| ARTHROv5_30654 |  | Nitrilase/cyanide hydratase | -1,71 | -0,09 | -0,56 | -2,42 | -0,16 | -0,27 | -2,44 | -0,74 | -0,35 |
| ARTHROv5_40491 | *speB* | putative agmatine ureohydrolase | -0,09 | 1,32 | 1,62 | -0,08 | 1,07 | 2,00 | -0,30 | 0,62 | 1,92 |
| ARTHROv5_40618 | *nrtD* | ABC Nitrate transport system | -1,73 | -0,84 | -0,61 | -2,01 | -1,39 | -0,19 | -1,30 | -2,14 | -0,65 |
| ARTHROv5_40619 | *nrtC* | ABC Nitrate transport system, ATP-binding protein | -1,65 | -0,72 | -0,54 | -1,89 | -1,18 | -0,21 | -1,26 | -1,89 | -0,62 |
| ARTHROv5_40620 | *nrtB* | ABC Nitrate transport system | -1,51 | -0,87 | -0,49 | -2,01 | -1,21 | -0,09 | -1,62 | -2,10 | -0,68 |
| ARTHROv5_40621 | *nrtA* | ABC Nitrate transport system | -1,49 | -0,63 | -0,94 | -1,93 | -1,31 | -0,43 | -2,25 | -2,65 | -1,01 |
| Amino-acids transport and metabolism |  |  |  |  |  |  |  |  |  |  |  |
| ARTHROv5_10389 | *aapJ* | General L-amino acid-binding periplasmic protein AapJ | -1,46 | -0,41 | -0,45 | -1,91 | -0,35 | -0,31 | -2,15 | -0,64 | -0,57 |
| ARTHROv5_30372 | *aapP* | amino-acid transporter subunit ; ATP-binding component of ABC superfamily | -0,41 | 0,30 | -0,19 | -1,22 | 0,22 | 0,09 | -2,35 | -0,19 | -0,16 |
| ARTHROv5_40660 | *aapQ* | amino-acid transporter subunit ; membrane component of ABC superfamily | -0,88 | -0,60 | -0,36 | -1,57 | -0,65 | -0,31 | -1,96 | -1,07 | -0,62 |
| ARTHROv5_30436 | *argG* | Argininosuccinate synthase | -2,83 | -0,78 | -1,44 | -3,63 | -1,13 | -0,92 | -3,31 | -2,33 | -1,39 |
| ARTHROv5_30675 | *argH* | Argininosuccinate lyase | -1,60 | -0,08 | -0,69 | -1,67 | -0,21 | -0,31 | -1,01 | -0,93 | -0,52 |
| ARTHROv5_60801 | *argJ* | Arginine biosynthesis bifunctional protein argJ | 0,01 | 0,22 | -0,18 | -0,52 | 0,46 | -0,33 | -0,78 | 1,10 | -0,53 |
| ARTHROv5_60579 | *iaaA* | L-asparaginase, Peptidase T2 | -0,61 | 0,10 | -0,76 | -1,09 | -0,02 | -0,36 | -2,01 | -0,76 | -0,53 |
| ARTHROv5_10485 | *livG* | leucine/isoleucine/valine transporter subunit | -3,46 | -1,20 | -0,37 | -3,36 | -1,94 | -0,04 | -3,07 | -2,29 | -0,13 |
| ARTHROv5_10486 | *livM* | leucine/isoleucine/valine transporter subunit | -3,56 | -1,73 | -0,81 | -4,07 | -2,28 | -0,07 | -4,19 | -2,90 | -0,89 |
| ARTHROv5_10487 | *livH* | leucine/isoleucine/valine transporter subunit | -3,54 | -1,31 | -0,96 | -4,23 | -2,06 | -0,36 | -4,10 | -2,95 | -0,93 |
| ARTHROv5_10488 | *livJ* | leucine/isoleucine/valine transporter subunit | -3,11 | -1,22 | -1,00 | -3,70 | -2,16 | -0,61 | -4,05 | -2,75 | -1,02 |
| ARTHROv5_40571 | *patA1* | Putative Subtilisin-like serine protease, PatA-like | -2,42 | -0,39 | -1,29 | -2,46 | -0,94 | -0,63 | -2,49 | -2,44 | -1,01 |
| ARTHROv5_40573 | *patA2* | Putative Subtilisin-like serine protease, PatA-like | -2,06 | -0,59 | -1,66 | -2,04 | -0,94 | -0,77 | -2,64 | -2,72 | -1,22 |
| ARTHROv5_40574 | *patB* | conserved hypothetical protein, PatB-like | -2,25 | -0,60 | -1,52 | -2,09 | -1,03 | -0,69 | -2,26 | -2,75 | -1,14 |
| ARTHROv5_40575 | *patC* | conserved hypothetical protein, PatC-like | -2,57 | -0,91 | -1,61 | -2,39 | -1,27 | -0,81 | -2,09 | -3,22 | -1,39 |
| Cell envelope biogenesis |  |  |  |  |  |  |  |  |  |  |  |
| ARTHROv5_30755 | *murA* | UDP-N-acetylglucosamine 1-carboxyvinyltransferase | -1,16 | -0,27 | -0,99 | -2,09 | -0,53 | -0,61 | -2,97 | -1,54 | -0,84 |
| ARTHROv5_30610 | *murB* | UDP-N-acetylenolpyruvoylglucosamine reductase | -2,02 | -0,75 | -0,99 | -2,58 | -0,91 | -0,95 | -2,67 | -1,64 | -1,21 |
| ARTHROv5_30611 | *murC* | UDP-N-acetylmuramate--L-alanine ligase | -1,99 | -0,84 | -1,17 | -2,82 | -1,06 | -1,16 | -3,22 | -1,71 | -1,50 |
| ARTHROv5_60380 | *murD* | UDP-N-acetylmuramoylalanine--D-glutamate ligase | -0,08 | 0,13 | -0,72 | 0,81 | 0,06 | -0,57 | 2,10 | 0,39 | -0,81 |
| ARTHROv5_40267 | *murE* | UDP-N-acetylmuramoyl-L-alanyl-D-glutamate:meso-d iaminopimelate ligase | -1,66 | 0,06 | -0,78 | -2,37 | 0,03 | -0,46 | -2,73 | -0,75 | -0,87 |
| ARTHROv5_30718 | *mrdA1* | transpeptidase involved in peptidoglycan synthesis | -0,96 | 0,23 | 0,23 | -1,40 | 0,18 | 0,28 | -0,81 | 0,02 | 0,27 |
| ARTHROv5_20021 | *pilT1* | Type IV pilus twitching motility protein | -1,00 | -0,25 | -0,43 | -1,67 | 0,02 | -0,18 | -1,67 | -0,26 | -0,69 |
