# Supplementary material for: Temporal Gene Expression of the Cyanobacterium Arthrospira in Response to Gamma Rays
Source: PLoS One. 2015 Aug 26;10(8):e0135565. doi: 10.1371/journal.pone.0135565 (PMC4550399; doi:10.1371/journal.pone.0135565)
Supplement: S4 Table — As input the fold changes of those genes are having Log2FC was equal or higher than 1 for up-regulated genes, and equal or lower than-1 for the down regulated ones and a p-value corrected for multiple testing lower than 0.05 in either one of the 9 conditions. (DOCX) [file pone.0135565.s005.docx]

| Recovery period | Gene name | Gene function | 800 T0H | 800 T2H | 800 T5H | 1600 T0H | 1600 T2H | 1600 T5H | 3200 T0H | 3200  T2H | 3200  T5H |
| --- | --- | --- | --- | --- | --- | --- | --- | --- | --- | --- | --- |
| Sulphur metabolism |  |  |  |  |  |  |  |  |  |  |  |
| ARTHROv5_40486 | *tauB* | ABC transport system for taurine, ATP-binding component | -0,05 | 1,33 | 1,52 | -0,14 | 0,91 | 2,06 | -0,42 | 0,31 | 1,72 |
| ARTHROv5_40487 | *tauC* | ABC transport system for taurine, permease component | 0,07 | 1,48 | 1,53 | 0,04 | 1,11 | 2,00 | -0,25 | 0,51 | 1,67 |
| ARTHROv5_40488 | *tauA* | ABC transport system for taurine, periplasmic component | 0,04 | 1,48 | 1,48 | 0,06 | 1,09 | 1,97 | -0,20 | 0,54 | 1,68 |
| Hydrogen production |  |  |  |  |  |  |  |  |  |  |  |
| ARTHROv5_41303 | *hoxW* | Putative Ni,FE-hydrogenase maturation factor | 0,95 | -0,15 | 0,52 | 1,24 | 0,08 | 0,73 | 1,47 | 0,07 | 0,62 |
| ARTHROv5_40489 | *hypB1* | GTP hydrolase involved in nickel liganding into hydrogenases | 0,22 | 1,72 | 2,08 | 0,18 | 1,27 | 2,57 | -0,03 | 0,70 | 2,35 |
| ARTHROv5_40490 | *hypA1* | hydrogenase expression/formation protein | -0,05 | 1,61 | 1,89 | -0,01 | 1,17 | 2,31 | -0,15 | 0,64 | 2,20 |
| Glutathione production |  |  |  |  |  |  |  |  |  |  |  |
| ARTHROv5_30647 | *gshB* | glutathione synthetase | 0,94 | 1,71 | 1,78 | 0,76 | 1,46 | 1,79 | 0,95 | 1,65 | 2,04 |
