# Supplementary material for: Temporal Gene Expression of the Cyanobacterium Arthrospira in Response to Gamma Rays
Source: PLoS One. 2015 Aug 26;10(8):e0135565. doi: 10.1371/journal.pone.0135565 (PMC4550399; doi:10.1371/journal.pone.0135565)
Supplement: S5 Table — As input the fold changes of those genes are having Log2FC was equal or higher than 1 for up-regulated genes, and equal or lower than-1 for the down regulated ones and a p-value corrected for multiple testing lower than 0.05 in either one of the 9 conditions. (DOCX) [file pone.0135565.s006.docx]

| Recovery Period | Gene name | Gene function | 800 T0H | 800 T2H | 800 T5H | 1600 T0H | 1600 T2H | 1600 T5H | 3200 T0H | 3200  T2H | 3200  T5H |
| --- | --- | --- | --- | --- | --- | --- | --- | --- | --- | --- | --- |
| ARTHROv5_10467 | *arhF* | conserved hypothetical protein | 3,74 | 4,36 | 4,32 | 2,46 | 4,19 | 4,43 | 3,29 | 4,34 | 4,36 |
| ARTHROv5_10468 | *arhE* | conserved hypothetical protein | 4,66 | 5,63 | 5,90 | 3,13 | 5,45 | 6,11 | 4,11 | 5,76 | 6,06 |
| ARTHROv5_10469 | *arhD* | conserved hypothetical protein | 3,81 | 4,28 | 4,02 | 2,77 | 4,14 | 4,25 | 3,21 | 4,25 | 4,23 |
| ARTHROv5_10471 | *arhB* | conserved hypothetical protein | 4,02 | 4,68 | 4,46 | 2,93 | 4,46 | 4,87 | 3,16 | 4,67 | 4,81 |
| ARTHROv5_10472 | *arhA* | putative ABC-type phosphate transport | 3,43 | 4,57 | 4,27 | 2,39 | 4,33 | 4,46 | 3,38 | 4,71 | 4,76 |
